# MELK prevents radiofrequency ablation-induced immunogenic cell death and antitumor immune response by stabilizing FABP5 in hepatocellular malignancies

**DOI:** 10.1186/s40779-024-00588-7

**Published:** 2025-01-27

**Authors:** Bu-Fu Tang, Wang-Ting Xu, Shi-Ji Fang, Jin-Yu Zhu, Rong-Fang Qiu, Lin Shen, Yang Yang, Qiao-You Weng, Ya-Jie Wang, Jia-Yi Ding, Xiao-Jie Zhang, Wei-Qian Chen, Li-Yun Zheng, Jing-Jing Song, Biao Chen, Zhong-Wei Zhao, Min-Jiang Chen, Jian-Song Ji

**Affiliations:** 1 Key Laboratory of Imaging Diagnosis and Minimally Invasive Intervention Research, Lishui Hospital, School of Medicine, Zhejiang University, the Fifth Affiliated Hospital of Wenzhou Medical University, Lishui, 323000 Zhejiang China; 2 Institute of Imaging Diagnosis and Minimally Invasive Intervention Research, the Fifth Affiliated Hospital of Wenzhou Medical University, Lishui, 323000 Zhejiang China; 3 Department of Radiology, Sir Run Run Shaw Hospital, School of Medicine, Zhejiang University, Hangzhou, 310016 China; 4 Department of Pulmonary and Critical Care Medicine, Regional Medical Center for National Institute of Respiratory Diseases, Sir Run Run Shaw Hospital, School of Medicine, Zhejiang University, Hangzhou, 310016 China; 5 Clinical College of the Affiliated Central Hospital, School of Medicine, Lishui University, Lishui, 323000 Zhejiang China

**Keywords:** Radiofrequency ablation, Liver cancer, Maternal embryonic leucine zipper kinase (MELK), Fatty acid-binding protein 5 (FABP5), Immune microenvironment, Nanoparticles

## Abstract

**Background:**

Radiofrequency ablation (RFA) is an efficient treatment with unlimited potential for liver cancer that can effectively reduce patient mortality. Understanding the biological process related with RFA treatment is important for improving treatment strategy. This study aimed to identify the critical targets for regulating the efficacy of RFA.

**Methods:**

The RFA treatment in hepatocellular carcinoma (HCC) tumor models in vivo, was analyzed by RNA sequencing technology. The heat treatment in vitro for HCC tumor cells was also constructed to explore the mechanism after RFA treatment in tumor cells. Nanoparticles with high affinity to tumor cells were applied as a new therapy to interfere with the expression of maternal embryonic leucine zipper kinase (MELK).

**Results:**

It was found that RFA treatment upregulated MELK expression, and MELK inhibition promoted RFA efficacy by immunogenic cell death and the antitumor response, including anti-tumoral macrophage polarization and increased CD8^+^ T cell cytotoxicity in HCC. Mechanically, MELK binds to fatty acid-binding protein 5 (FABP5), and affects its ubiquitination through the K48R pathway to increase its stability, thereby activating protein kinase B (Akt)/mammalian target of rapamycin (mTOR) signaling axis to weaken the RFA-mediated antitumor effect. In addition, the synthesis of arginylglycylaspartic acid (RGD)-lipid nanoparticles (LNPs) targeting tumor cell-intrinsic MELK enhanced RFA efficacy in HCC.

**Conclusion:**

MELK is a therapeutic target by regulating RFA efficacy in HCC, and targeting MELK via RGD-LNPs provides new insight into improving RFA efficacy in HCC clinical treatment and combating the malignant progression of liver cancer.

**Supplementary Information:**

The online version contains supplementary material available at 10.1186/s40779-024-00588-7.

## Background

Primary liver cancer is one of the most common malignant tumors of the digestive system, ranked the third leading cause of tumor-related death worldwide [1]. Nearly 90% of primary liver cancers are hepatocellular carcinoma (HCC) [2]. Surgery is currently the only approach to cure early liver cancer with limited indication. Radiofrequency ablation (RFA) is a prospective therapeutic technique, that has a broader range of indications, fewer complications, and significantly shorter overall hospital stays than surgical treatment [3, 4]. It causes ions to shock and heat through radiofrequency electrodes, raising the temperature of the local tissue to kill tumor cells [5]. In addition, previous study has indicated that RFA activates the immune system to induce the killing of distal tumors, called the “abscopal effect” [6]. However, the effect of RFA is not sustainable and is often accompanied by relapse [6]. Therefore, how to amplify and sustain RFA’s therapeutic effect is a critical research topic.

Immunogenic cell death (ICD) is a regulatory cell death mode that specifically causes an immune response to dead or dying tumor cell antigens, including induced damage-associated molecular patterns, tumor-associated or tumor-specific antigens, and compositive-damage-associated molecular patterns (cDAMP) [7, 8]. Cytokines [such as interleukin (IL)-6] and chemokines [such as C-X-C motif chemokine ligand 10 (CXCL10)] are crucial to injury induction. In addition, cDAMP is involved in high mobility group box 1 (HMGB1) and ATP release. The immune system absorbs the cell debris and intracellular material (such as organelles, tumor antigens, and injury-associated molecular patterns including HMGB1) released due to the vast cell death caused by the heat shock transmission from RFA. As an antigen source of the immune system, it is taken up by dendritic cells to stimulate immune activation. Solid tumors are often difficult to treat and resistant to drugs due to dense tumor tissue, while RFA can vigorously promote tumor microenvironment (TME) remodeling. Studies have shown that RFA can activate the immune system by inducing ICD, which is believed to be the key to producing the antitumor response [9, 10].

Maternal embryonic leucine zipper kinase (MELK), also known as murine protein serine-threonine kinase 38 (MPK38), is a member of the adenosine monophosphate (AMP)-related serine-threonine kinase family involved in various physiological and pathological processes, such as cell proliferation, cell cycle regulation, and apoptosis inhibition [11]. Many recent studies have shown that MELK is highly expressed in various cancers, such as lung, breast, and gastric cancers [12–15]. Furthermore, RNA interference-mediated *MELK* knockdown has confirmed that MELK expression is vital for cancer cell proliferation and survival. *MELK* depletion slowed or stopped the proliferation and invasion of these and other cancer cell lines. Our previous research also found that MELK is important in colitis-propelled carcinogenesis, which is closely associated with immune regulation [16]. In addition, MELK expression was found to be abnormally high in liver cancer tissues, which is crucial for early liver cancer recurrence [17]. However, whether MELK affects RFA-induced tumor inhibition or immune microenvironment changes in liver cancer is unclear, and its specific role and regulatory mechanism remain unclear.

Fatty acid-binding protein 5 (FABP5) is a 15 kD small molecule protein mainly involved in fatty acid metabolism in the cytoplasm and is also a cytoplasmic transporter of oleic acid. Recent study has found that FABP5 plays a role in metabolic diseases and is crucial in the progression of various cancers, promoting cell proliferation, migration, and invasion [18]. Therefore, its expression closely relates to poor prognoses of cancers, such as liver, breast, and colorectal cancers [19, 20]. *FABP5* is considered an important oncogene in liver cancer progression [21]. FABP5 has been found to regulate antitumor immunity and affects HCC progression [22]. FABP5 expression is also closely associated with regulating programmed cell death, including apoptosis and autophagy in cancer [23, 24]. However, the role of FABP5 in regulating RFA-mediated ICD and immune activation remains unclear.

It is well-known that the greatest difficulty facing RFA in treating liver cancer is the inevitable tumor resistance to RFA. Therefore, there is an urgent need to explore the regulatory mechanisms of RFA resistance to develop new therapeutic strategies for HCC. This study investigated the role of the TME in RFA tumor treatment and elucidated the corresponding regulatory mechanism. Therefore, there is an unmet need to implement MELK intervention therapy to enhance RFA response in HCC. The wide application of nanomaterials provides a giant platform for cancer treatment [25]. We also report a platform targeting small interfering RNA (siRNA) nanoparticles designed to interfere with MELK expression to enhance RFA efficacy.

## Materials and methods

### Animal models

Six to eight weeks old C57BL/6 (*n* = 127) or nude male mice (*n* = 20) were purchased from Shanghai Laboratory Animal Center (Shanghai, China), and were housed in SPF criteria, be raised under (24 ± 2) °C, 40–70% relative humidity, 12-h light and 12-h dark cycle, and prepared for animal trials.

Two subcutaneous tumor-bearing models were established. Every group contained at least 5 mice. One was created by subcutaneously injecting 1 × 10^6^/100 µl of Hepa1-6 cells into the medial left lower extremity of 8-week-old male C57BL/6 mice. The second was created by subcutaneously injecting 1 × 10^6^/100 µl of HCC-LM3 or Hepa1-6 cells into the left medial lower extremity of 8-week-old male nude mice. When the tumor size reached about 100 mm^3^, an 18G bipolar ablation needle head was placed into the tumor tissue, the radiofrequency tail needle was connected to the radiofrequency transmitter, the radiofrequency temperature was set to 60 ℃, and the RFA was repeated for 2 min.

An orthotopic liver cancer model was established in a sterile environment by removing the subcutaneous tumors from mice and transplanting 1 mm^3^ into the liver parenchyma of mice, and sacrificing until day 21 [26]. Tumor volume = (1/2 × long diameter) × short diameter^2^. The diameters of the tumor were measured by a caliper. The tumor tissues originating from Hepa1-6-luciferase cell lines were constructed as in situ HCC tumors, which were detected through promising bioluminescence imaging. Mice with tumors larger than 20 mm on the longest axis or with a volume greater than 2 cm^3^ were euthanized [15]. All animal experiments were performed according to protocols approved by the Institutional Animal Care and Use Committee of Wenzhou Medical University (wydw2023-0452).

### Differentially expressed genes (DEGs) and enrichment analyses

The mRNA sequencing data and clinical information of patients with HCC were downloaded from open databases: The Cancer Genome Atlas (TCGA) and International Cancer Genome Consortium (ICGC) databases. Fresh tumor tissue samples, which were hypodermic implants of Hepa1-6 HCC cells, were collected from animal models treated with RFA for 24 h and those treated with the sham operation. The relative gene expression matrix was sequenced, and DEG analysis was performed using the R statistical software’s limma package [adjusted *P*-value < 0.05 and |log_2_ fold change (FC)|> 1]. The expression matrices for HCC and para-cancer tissues in the TCGA-liver HCC (LIHC) and ICGC-Japanese (JP)-HCC cohorts were analyzed using R’s limma package (adjusted *P*-value < 0.05 and |log_2_ FC|> 1). Gene Ontology (GO) and Kyoto Encyclopedia of Genes and Genomes (KEGG) enrichment analyses were used to functionally annotate DEGs and explore the functional and regulatory signaling pathways associated with RFA (adjusted *P*-value < 0.05).

### Immunofluorescence (IF) and immunohistochemistry (IHC)

The tissue sections of HCC tumors were dewaxed, hydrated, and antigen repaired. For IF, the tissue section was placed in the diluted primary antibody at 4 °C overnight, then incubated with 488/576 goat anti-rabbit/rat IgG (Invitrogen/Thermo Fisher Scientific, USA) at room temperature for 2 h. Finally, 4′,6-diamidino-2-phenylindole (DAPI) was incubated for 15 min to stain the nuclear DNA. For IHC, the tissue sections of HCC tumors were placed in 3% hydrogen peroxide in the dark for 20 min. The tissue sections were incubated with the horseradish peroxidase of the corresponding species at room temperature for 2 h. Then, the tissue sections were incubated with 3,3’-diaminobenzidine for color development. The IHC score used in this study was calculated by the ImageJ software based on the intensity and positive area as described previously [15, 27]. The specific staining intensity was graded as follows: 0 (negative), 1 (low positive), 2 (positive), 3 (high positive). Then, this value was used in the formula: IHC score = (% of high positive cells × 3) + (% of positive cells × 2) + (% of low positive cells × 1). All the antibodies were detailed in Additional file 1: Table S1.

### Cell culture and cell hyperthermia administration

This study used the Hepa1-6, SK-HEP1, HCC-LM3, LO2, and 293 T cell lines purchased from the American Type Culture Collection Cell Bank and confirmed by short tandem repeat analysis. Human HCC cell line SK-HEP1, highly metastatic human HCC cell line HCC-LM3, and murine HCC cell line Hepa1-6 were cultured in Dulbecco’s modified Eagle’s medium supplemented with 10% fetal bovine serum and 1% penicillin and streptomycin in an incubator at 37 ℃ and 5% CO_2_. Liver cancer SK-HEP1 and HCC-LM3 cells were heated in a water bath at 47 ℃ for 10 min to simulate in vitro thermal ablation.

### Flow cytometry

The cell suspension was prepared, and then the antibody was added for 15 min. Then, using a flow cytometer, the blank was used to draw the door, the voltage was adjusted, adhesion was removed, and the Flowjo software was used for subsequent experimental analysis. All the antibodies were detailed in Additional file 1: Table S2. FITC-Annexin V was used in conjunction with PI through flow cytometry to distinguish between living cells, early and late apoptotic cells, and dead cells. And the flow cytometry divided into different cell clusters including tumor-associated macrophage (TAM) (CD45^+^F4/80^+^CD11b^+^), CD8^+^ T cells (CD8^+^CD45^+^), M1 macrophages (CD16/32^+^F4/80^+^), and M2 macrophages (CD206^+^F4/80^+^) in tumor tissues for subsequent analysis.

### Small hairpin RNA (shRNA) construction

MELK-specific sequences with experimentally verified good knockdown efficiencies were downloaded from the official Sigma-Aldrich website. The shRNA sequences used in this study are listed in Additional file 1: Table S3.

### Protein–protein interaction docking model

FABP5 (PDB: 4LKP) was selected as ligand and MELK (PDB: 4IXP) as receptor for protein and protein docking. The HDOCK web service was used for docking, with default parameters (http://hdock.phys.hust.edu.cn/). Exported PDB files were used for job submission.

### Nanomaterials preparation

Lipid nanoparticles (LNPs) were created using a rapid mixing technique based on the principle of combining anions and ions. Cationic liposomes can be positively electrically bound to hydrogen ions in an acidic buffer environment (pH = 4.0) that ionizes the insoluble cationic lipids in the membrane to generate oil phases. Metastable particles in these oil phases continue to fuse until the LNP surface is coated with cationic lipids. In this study, siRNA-cyanine 5.5 (Cy5.5) is sandwiched between lipid layers and forms part of the LNP core, which is released from the LNPs and interferes with RNA in an acidic environment. In this study, siMELK (siRNA targeting MELK)-Cy5.5 and siNC (control) are inserted in LNPs, shown in Additional file 1: Table S3. A transmission electron microscope (TEM) was used to monitor the quality of LNPs, including the diameter and the morphology. We dosed mice with LNPs containing siRNA at therapeutically relevant concentrations through tail vein injection and inspected their function. Other materials and methods are shown in Additional file 1: Materials and methods.

### Statistical analysis

All experiments conducted in this study were independently repeated 3 times. All quantitative data are normally distributed and presented as the mean ± standard error of the mean. The *t*-test was used to compare the differences between two groups. Variables were compared between more than two groups using a one-way analysis of variance followed by Tukey’s post hoc tests or multi-comparisons. Results with a *P* < 0.05 were considered statistically significant. All cell experiments were independently repeated three times.

## Results

### RFA drives immune cell infiltration in mice hepatoma tissue

**Fig. 1:**
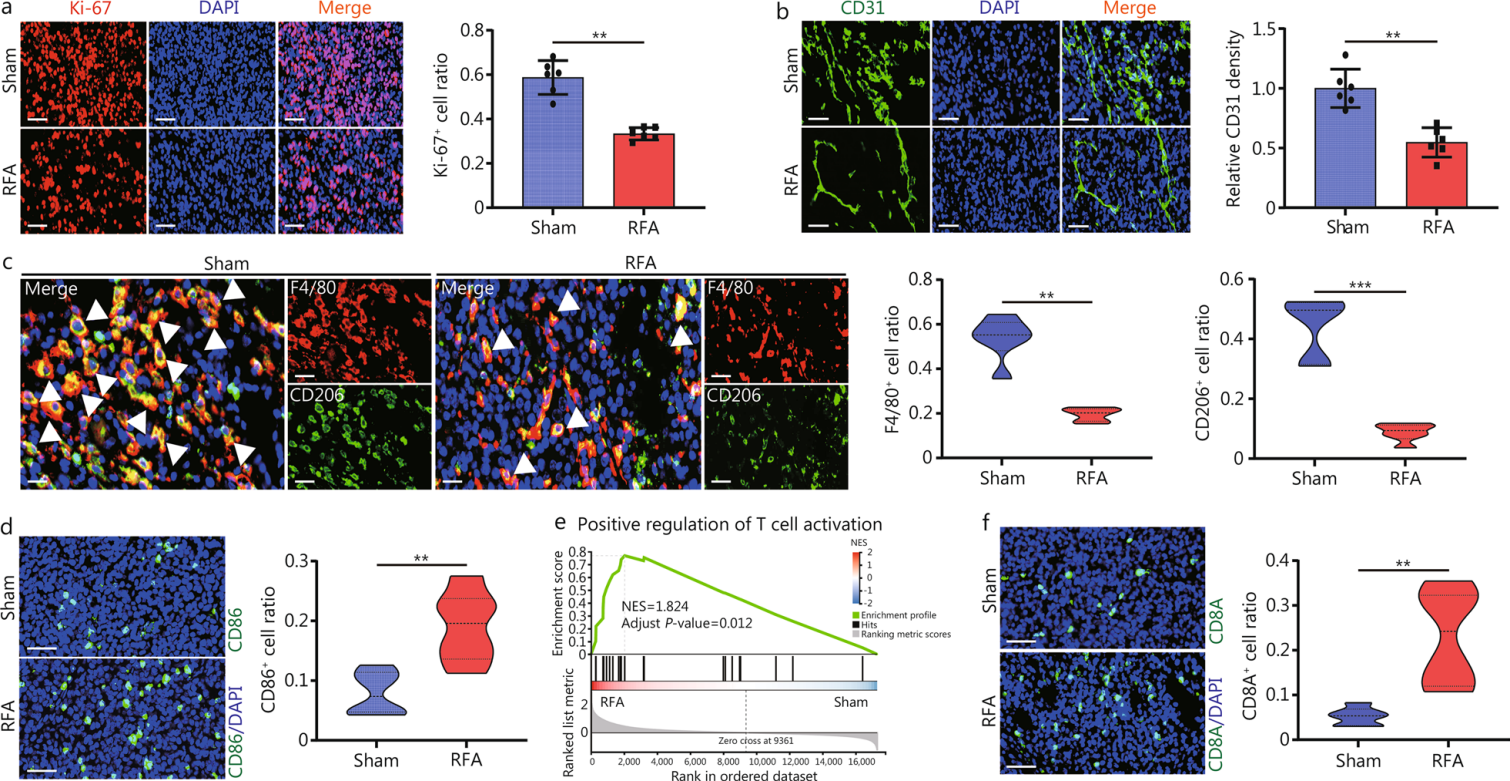
RFA inhibits tumor progression and induces immune cell infiltration. IF-based expression levels and corresponding positive ratio of Ki-67 (**a**) and CD31 (**b**) in Hepa1-6 tumor tissues. Ki-67 is in red, CD31 is in green, DAPI is in blue. **c** IF-based F4/80 and CD206 protein levels in tumors after sham or RFA treatment. The ratio of F4/80^+^ or CD206^+^ cells in tumor tissues after sham or RFA treatment. White arrows indicate the co-labeled cells of F4/80^+^ and CD206^+^. **d** IF-based CD86 protein levels in liver tumors after sham or RFA treatment. The ratio of CD86^+^ cells in tumor tissues after sham or RFA treatment. **e** Gene set enrichment analysis (GSEA) analysis shows the relationship between positive regulation of T cell activation pathway and RFA treatment. **f** IF-based CD8A^+^ expression in Hepa1-6 tumor tissues. Scale bar = 20 μm. ^**^P < 0.01, ^***^P < 0.001. RFA radiofrequency ablation, DAPI 4′,6-diamidino-2-phenylindole, NES normalized enrichment score

RFA has been shown to cure tumors by inducing cell death in the ablated areas and inhibiting tumor growth in non-ablated tumor areas by stimulating antitumor immunity [28, 29]. We constructed a mouse ablation model by transplanted syngeneic tumor cells to investigate immune microenvironment remodeling (Additional file 1: Fig. S1a). It showed that RFA inhibited the growth of HCC tumors (Additional file 1: Fig. S1b). Significant differences in gene expression after RFA treatment were shown in the volcano plots (Additional file 1: Fig. S1c). The enriched signaling pathway demonstrated that RFA treatment was closely related to the TME and tumor growth (Additional file 1: Fig. S1d–e). Furthermore, RFA treatment inhibited tumor proliferation and angiogenesis (Fig. 1a, b), but promoted tumor cell apoptosis (Additional file 1: Fig. S1f). Moreover, the immune system was activated by RFA treatment, with M1 polarization promotion and M2 polarization inhibition (Fig. 1c, d). CD206, the M2 type of macrophage marker was significantly downregulated after RFA treatment while CD86, the M1 type of macrophage marker was upregulated. Moreover, gene set enrichment analysis (GSEA) and CD8A^+^ detection noted that RFA exerts antitumor effects directly on tumor cell death and possibly on TME remodeling (Fig. 1e, f).

### MELK is highly expressed in HCC tissues after RFA treatment and functions as an independent risk factor in patients with HCC

**Fig. 2:**
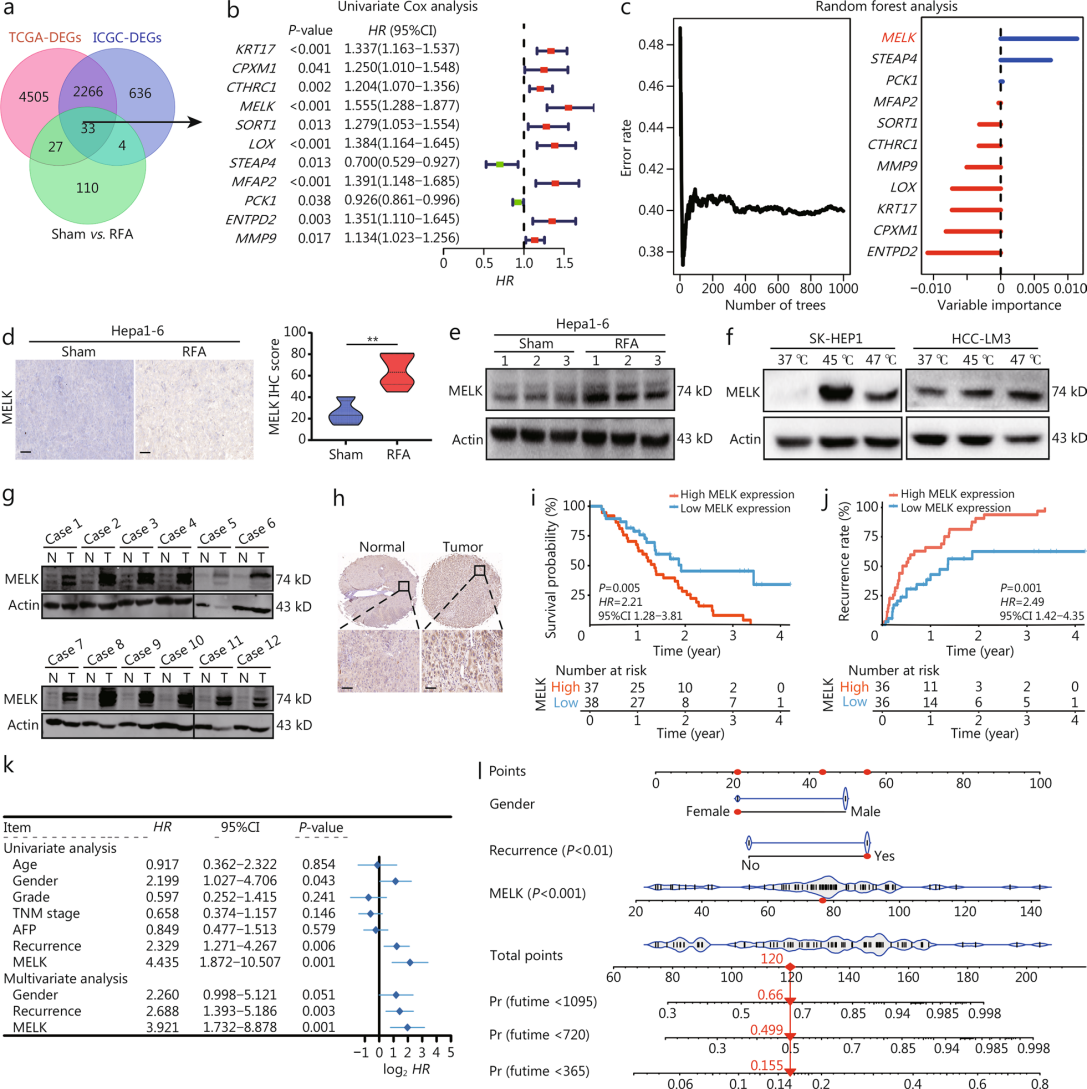
RFA treatment increased MELK expression, an independent risk factor in patients with HCC. **a** A Venn diagram of the common DEGs of the TCGA-LIHC cohort, the ICGC-JP cohort, and the RFA processing groups. **b** A univariate Cox regression analysis shows the potential regulatory genes related to liver cancer prognosis. **c** The random forest algorithm shows the important genes related to liver cancer prognosis. **d** IHC shows MELK expression in Hepa1-6 liver cancer tissues after RFA or sham treatment and relative MELK IHC scores. **e** Western blotting shows MELK expression in Hepa1-6 liver cancer tissue after RFA or sham treatment (*n* = 3). **f** Western blotting shows MELK expression in SK-HEP1 and HCC-LM3 cells after heat treatment. Western blotting (**g**) and IHC (**h**) show MELK expression in human liver cancer and adjacent tissues. **i** Survival probability of patients with liver cancer and different IHC scores of MELK. **j** The recurrence rate of patients with liver cancer and different IHC scores of MELK. **k** A forest plot shows the independent risk factors for liver cancer. **l** A nomogram of the assessment of 1-, 2-, and 3-year overall survival of patients with a predicted model. Scale bar = 20 μm. ^**^P < 0.01. RFA radiofrequency ablation, DEG differentially expressed gene, HCC hepatocellular carcinoma, MELK maternal embryonic leucine zipper kinase, IHC immunohistochemistry, AFP alpha-fetoprotein, TCGA The Cancer Genome Atlas, LIHC TCGA-liver HCC, ICGC International Cancer Genome Consortium, JP Japanese, Pr Probability

It identified 33 common DEGs of the two liver cancer cohorts and RFA-treated HCC samples (Fig. 2a). *MELK*, the oncogenic gene, is a significant DEG and upregulated after RFA treatment (Fig. 2b–e). In addition, MELK expression was also upregulated after heating treatment (Fig. 2f, Additional file 1: Fig. S2a, b). Then we found that MELK expression was significantly higher in HCC tissues than that in para-cancerous tissues (Fig. 2g, h, Additional file 1: Fig. S2c–f). The high expression of MELK in HCC tumor tissue predicted a decrease in survival and an increase in recurrence rate (Fig. 2i, j). Patients with HCC of high MELK expression had shorter overall survival and disease-free survival (Additional file 1: Fig. S2g, h). Univariate and multivariate analyses and Cox regression nomogram calculation of risk scores for all patients suggested that MELK was an independent risk factor for poor HCC prognosis (Fig. 2k, l).

### *MELK* knockdown enhances HCC sensitivity to heat treatment and RFA efficacy

To mimic thermal ablation by RFA treatment in vitro, we heated SK-HEP1 and HCC-LM3 liver cancer cells in a 47 °C water bath for 10 min. It showed that heat-up or *MELK* knockdown induced cell death and combined treatment enlarged this phenomenon. Besides, heat-up or *MELK* knockdown inhibited cell viability, exhibiting high expression of Eth-1 and low expression of Cal-AM, and also inhibited proliferation, showing low detection of EDU staining, and combined treatment enlarged those effects in hepatoma cell lines (Additional file 1: Fig. S3a–d). A xenograft tumor model of HCC-LM3 with RFA treatment was constructed to determine whether MELK expression regulates sensitivity to RFA treatment (Additional file 1: Fig. S3e). Tumor size, volume, and weight were significantly lower in RFA-treated and MELK knockdown groups than those in sham group and were lowest in group combining *MELK* knockdown with RFA treatment (Additional file 1: Fig. S3f–h). It showed that RFA treatment upregulated the expression of MELK in tumor tissues. *MELK*-knockdown exhibited high efficiency which also antagonism the up-level of MELK induced by RFA treatment (Additional file 1: Fig. S3i).

Mechanically, *MELK* knockdown or RFA treatment inhibited tumor proliferation and migration but promoted apoptosis, the combination enlarged this effect (Additional file 1: Fig. S3j). More importantly, phosphorylation of the transcription factor signal transducer and activator of transcription 3 (p-STAT3) is a biomarker of tumor growth and metastasis, which was decreased after RFA treatment after MELK deprivation and RFA treatment. These results suggest that MELK is involved in the RFA regulatory process and that *MELK* knockdown can effectively improve RFA efficacy in liver cancer.

### *MELK* knockdown promotes RFA-induced hepatoma cell apoptosis and immunogenic death

**Fig. 3:**
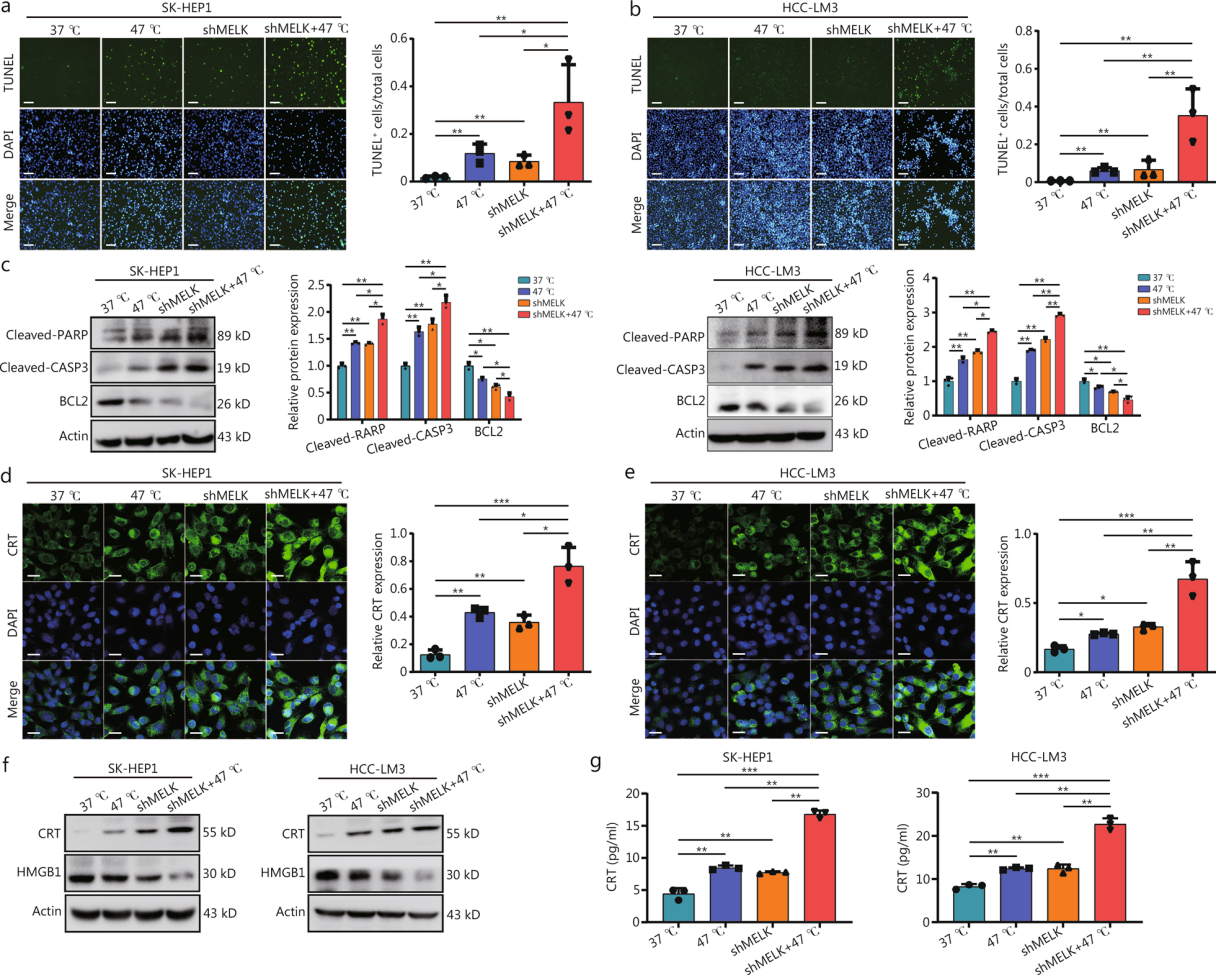
*MELK* knockdown promotes RFA-induced apoptosis and immunogenic death in hepatoma cells. TUNEL staining shows apoptosis levels in SK-HEP1 (**a**) and HCC-LM3 (**b**) cells. TUNEL is in green, DAPI is in blue. Statistical map of TUNEL-positive cells (*n* = 3). **c** Western blotting shows cleaved-RARP, cleaved-CASP3, BCL2, and actin levels in SK-HEP1 and HCC-LM3 cells with different treatments (*n* = 3). Representative IF images show CRT expression in SK-HEP1 (**d**) and HCC-LM3 (**e**) cells with different treatments (*n* = 3). CRT is in green, DAPI is in blue. **f** Western blotting shows CRT, HMGB1, and actin expression in SK-HEP1 and HCC-LM3 cells. **g** ELISA results show the secretion level of CRT in SK-HEP1 and HCC-LM3 cells (*n* = 3). Scale bar = 20 μm. ^*^P < 0.05, ^**^P < 0.01, ^***^P < 0.001. MELK maternal embryonic leucine zipper kinase, DAPI 4′,6-diamidino-2-phenylindole, RFA radiofrequency ablation, RARP poly (ADP-ribose) polymerase, CASP3 Caspase-3, BCL2 B-cell leukemia/lymphoma 2, HCC hepatocellular carcinoma, IF immunofluorescence, IHC immunohistochemistry, CRT calreticulin, HMGB1 high mobility group box 1

The in vitro model was highly consistent with the animal model with RFA treatment. Ablation or *MELK*-deleted resulted in cell apoptosis, and combined treatment has the best effects (Fig. 3a-b, Additional file 1: Fig. S4a). Cleaved-poly (ADP-ribose) polymerase (RARP), cleaved-CASP3, and B-cell leukemia/lymphoma 2 (BCL2) levels were also detected. It was found that both shMELK and high temperature increased the expression of pro-apoptotic cleaved-PARP/-CASP3 and inhibited the expression of anti-apoptotic BCL2, resulting the programmed cell death (Fig. 3c).

Previous studies showed that RFA triggers ICD, which is critical in tumor progression [10, 30]. Calreticulin (CALR/CRT) and HMGB1 expression (Fig. 3d–f), especially that the upregulated extracellular expression of CRT and HMGB1 (Fig. 3g, Additional file 1: Fig. S4b), indicated that while heat treatment and *MELK* knockdown alone can effectively induce liver cancer cell apoptosis and ICD, combining them has an even more pronounced effect, which may partly explain why *MELK* knockdown can further enhance the inhibition of tumor cell growth caused by thermal ablation in liver cancer.

### *MELK* knockdown enhances RFA-induced antitumor immune effects in HCC

A xenograft tumor model was constructed with Hepa1-6 cells to explore the role of MELK in regulating the sensitivity of liver cancer to RFA (Additional file 1: Fig. S5a). MELK or RFA treatment inhibited the progression of liver tumors, and combined group enhanced this anti-tumor effect (Additional file 1: Fig. S5b–e). We used flow cytometry to investigate further the infiltration of immune cells in the tumor immune microenvironment (Additional file 1: Fig. S5f). TAM infiltration into mouse liver cancers was inhibited by RFA treatment or *MELK* knockdown, with increased M1 macrophages, decreased M2 macrophages in the TAM cluster, and increased CD8^+^ T cell infiltration. Moreover, combining *MELK* knockdown with RFA treatment enhanced those effects (Additional file 1: Fig. S5f). And the proportion of proliferating cells and macrophages were significantly inhibited (Additional file 1: Fig. S5g, h), with a stronger effect observed when *MELK* knockdown was combined with RFA treatment. It showed that RFA or *MELK*-knockdown altered the immune condition of hepatoma cancer.

In addition, a multiplex cytokine array showed that C–C motif chemokine ligand 2 (CCL2) secretion was decreased, and CXCL10, IL-1β, tumor necrosis factor-α (TNF-α), IL-2, CXCL11, CCL5, and IFN-γ secretion was increased in group combining *MELK* knockdown with RFA treatment (Additional file 1: Fig. S5i, j). It also confirmed that combining *MELK* knockdown with RFA treatment could induce antitumor immune activation.

To further explore whether the RFA-induced anti-proliferative effect was closely associated with MELK expression, Hepa1-6 tumors from the 4 groups in Additional file 1: Fig. S5b were cut into small (1 mm^3^) cubes and inoculated into the liver parenchyma to construct an orthotopic liver tumor model (Additional file 1: Fig. S5k). While in situ HCC progression was inhibited in both the RFA treatment and *MELK* knockdown groups, the effects were greater in group combining *MELK* knockdown with RFA treatment (Additional file 1: Fig. S5l–n). RFA treatment or *MELK* knockdown exhibited CD206 expression was downregulated, CD8 and CD86 positive cells were increased, and the combined treatment amplifies this effect (Additional file 1: Fig. S6a–c). The degree of ICD intratumor increased with RFA or *MELK*-deleted treatment, presenting HMGB1 downregulated and CRT upregulated, and the combined treatment also showed the enlarged ICD effect (Additional file 1: Fig. S6d–f). These results demonstrate that targeting MELK regulates the sensitivity of liver cancer to RFA, which is closely associated with immune microenvironment remodeling.

### Tumor cell-intrinsic MELK enhanced the protein kinase B (Akt)/mammalian target of rapamycin (mTOR) signal axis and the interaction with FABP5 in HCC

**Fig. 4:**
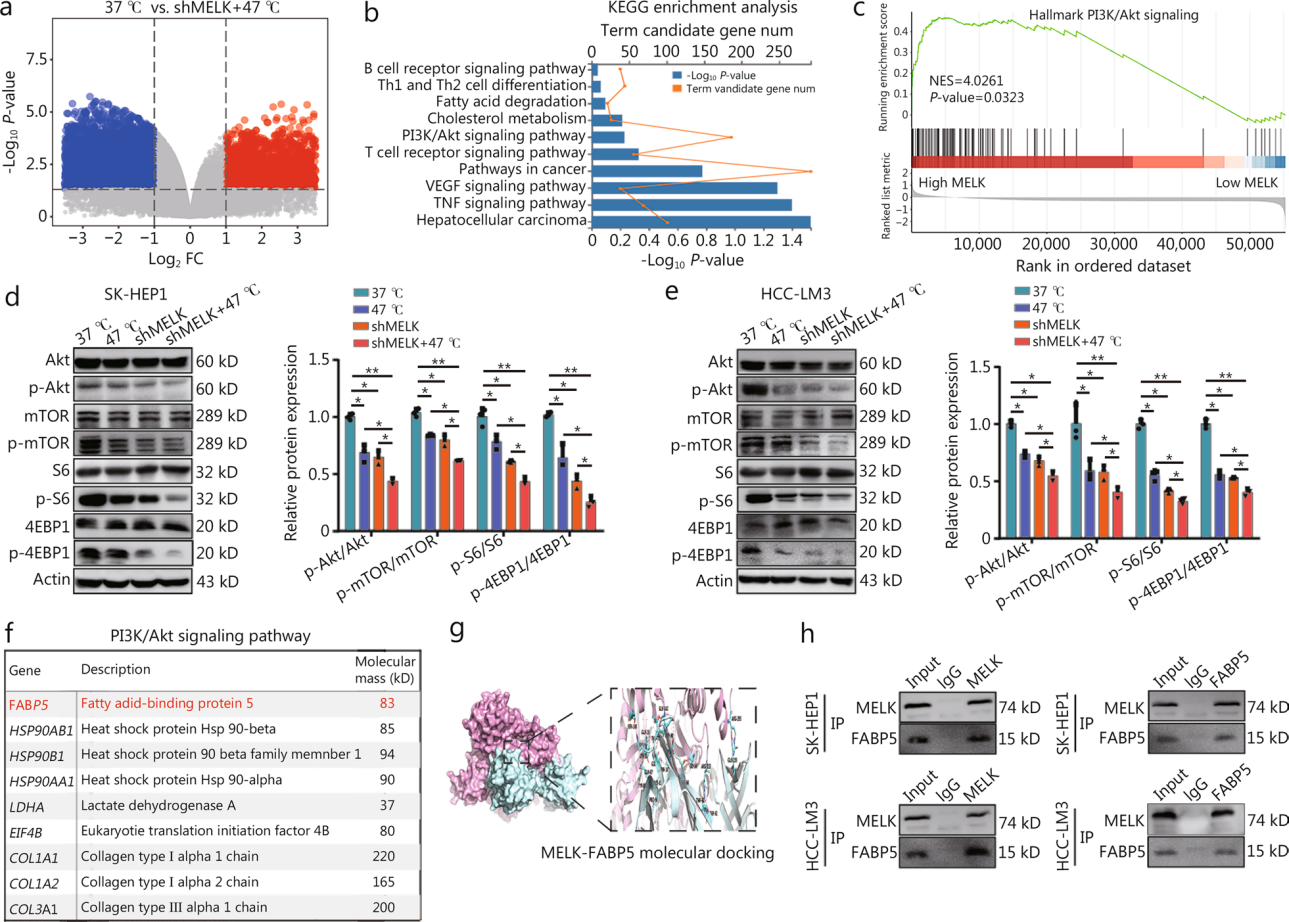
Tumor cell-intrinsic MELK enhanced the PI3K/Akt/mTOR signal axis and FABP5 interaction. **a** A volcano plot shows DEGs between the control (37 ℃) and combined shMELK with 47 ℃ treatments in SK-HEP1 cells. **b** KEGG enrichment analysis shows *MELK* knockdown and hyperthermia co-treatment-related signaling pathways in SK-HEP1 cells. **c** Gene set enrichment analysis (GSEA) analysis shows the hallmark gene sets between high and low MELK expression in liver cancers in the TCGA-LIHC cohort. Immunoblotting shows Akt/p-Akt, mTOR/p-mTOR, S6/p-S6, 4EBP1/p-4EBP1, and actin in SK-HEP1 (**d**) and HCC-LM3 (**e**) cells. A histogram of relative p-Akt/Akt, p-mTOR/mTOR, p-S6/S6, and p-4EBP1/4EBP1 expression (*n* = 3). **f** Protein schematics of PI3K/Akt-related pathways. **g** A schematic representation of MELK and FABP5 molecular docking. **h** Western blotting shows the interaction between MELK and FABP5 in SK-HEP1 and HCC-LM3 cells. ^*^P < 0.05, ^**^P < 0.01. MELK maternal embryonic leucine zipper kinase, DEGs differentially expressed genes, KEGG Kyoto Encyclopedia of Genes and Genomes, VEGF vascular endothelial growth factor, TNF tumor necrosis factor, NSE normalized enrichment score, PI3K phosphoinositide 3-kinase, Akt protein kinase B, mTOR mammalian target of rapamycin, S6 ribosomal protein S6, 4EBP1 eukaryotic translation initiation factor 4E binding protein 1, LC–MS liquid chromatography-mass spectrometry, FABP5 fatty acid-binding protein 5

Our findings showed that MELK is a crucial factor in liver cancer progression and its sensitivity to RFA. However, the downstream regulatory mechanisms through which MELK affects RFA efficacy and residual cancer progression remain unknown. To identify the potential mechanism of MELK regulating liver cancer sensitivity to RFA, liver cancer SK-HEP1 cells were subjected to *MELK* knockdown treatment and heat treatment at 47 ℃ before RNA sequence analysis, with DEGs visualized in a volcano map (Fig. 4a). The DEGs were explored in GO and KEGG enrichment analyses. DEGs were mainly enriched in pathways related to cell death, the immune response, and tumor signal transduction, such as protein ubiquitination (Ub), protein phosphorylation, TOR signaling pathway, phosphoinositide 3-kinase (PI3K)-Akt signaling pathway, and T cell receptor signaling pathway (Fig. 4b, Additional file 1: Fig. S7a). Therefore, we hypothesized that *MELK* knockdown in HCC cells would likely play a synergistic antitumor role through these signaling pathways with thermal ablation in HCC. Interestingly, GSEA also identified the “PI3K/Akt signaling pathway”, which is also an immune regulation signaling pathway (Fig. 4c).

Western blotting was used to measure the key proteins in the PI3K/Akt/mTOR signaling pathway to support this hypothesis. Figure 4d, e shows that the phosphorylation levels but not the expression levels of key proteins in this signaling pathway were decreased by 47 ℃ treatment and *MELK* knockdown compared with control group: p-Akt (Ser473), p-mTOR (Ser248), phosphorylation of ribosomal protein S6 (p-RPS6/p-S6; Ser240/244), and phosphorylation of eukaryotic translation initiation factor 4E binding protein 1 (p-EIF4EBP1/p-4EBP1; Ser65). Combining *MELK* knockdown with 47 ℃ decreased the phosphorylation levels of these proteins more than heat treatment alone without affecting their overall protein levels. This finding also suggests that *MELK* knockdown enhances HCC sensitivity to RFA by interfering with PI3K/Akt/mTOR signaling.

Next, we analyzed MELK interacting proteins using LC–MS (Fig. 4f). Additional file 1: Fig. S7b shows that FABP5 is a critical protein interacting with MELK. We also computationally simulated the molecular anchoring diagram of MELK and FABP5 (Fig. 4g) and created an interaction diagram for FABP5 and MELK in the background based on Western blotting in SK-HEP1 and LM3 liver cancer cells (Fig. 4h). It has been reported that FABP5 has been previously shown to activate PI3K/Akt/mTOR signaling [31, 32]. KEGG enrichment analysis (Additional file 1: Fig. S7c) was consistent with the results in Fig. 4b. The proteins binding to MELK were closely associated with the “PI3K/Akt signaling pathway” and “mTOR signaling pathway” (Additional file 1: Fig. S7c). These results suggest that MELK activates the PI3K/Akt/mTOR signaling axis in HCC cells and influences their thermal sensitivity, with downstream signaling potentially regulated by the interaction between MELK and FABP5.

### MELK decreased FABP5 Ub to maintain its stability in HCC

**Fig. 5:**
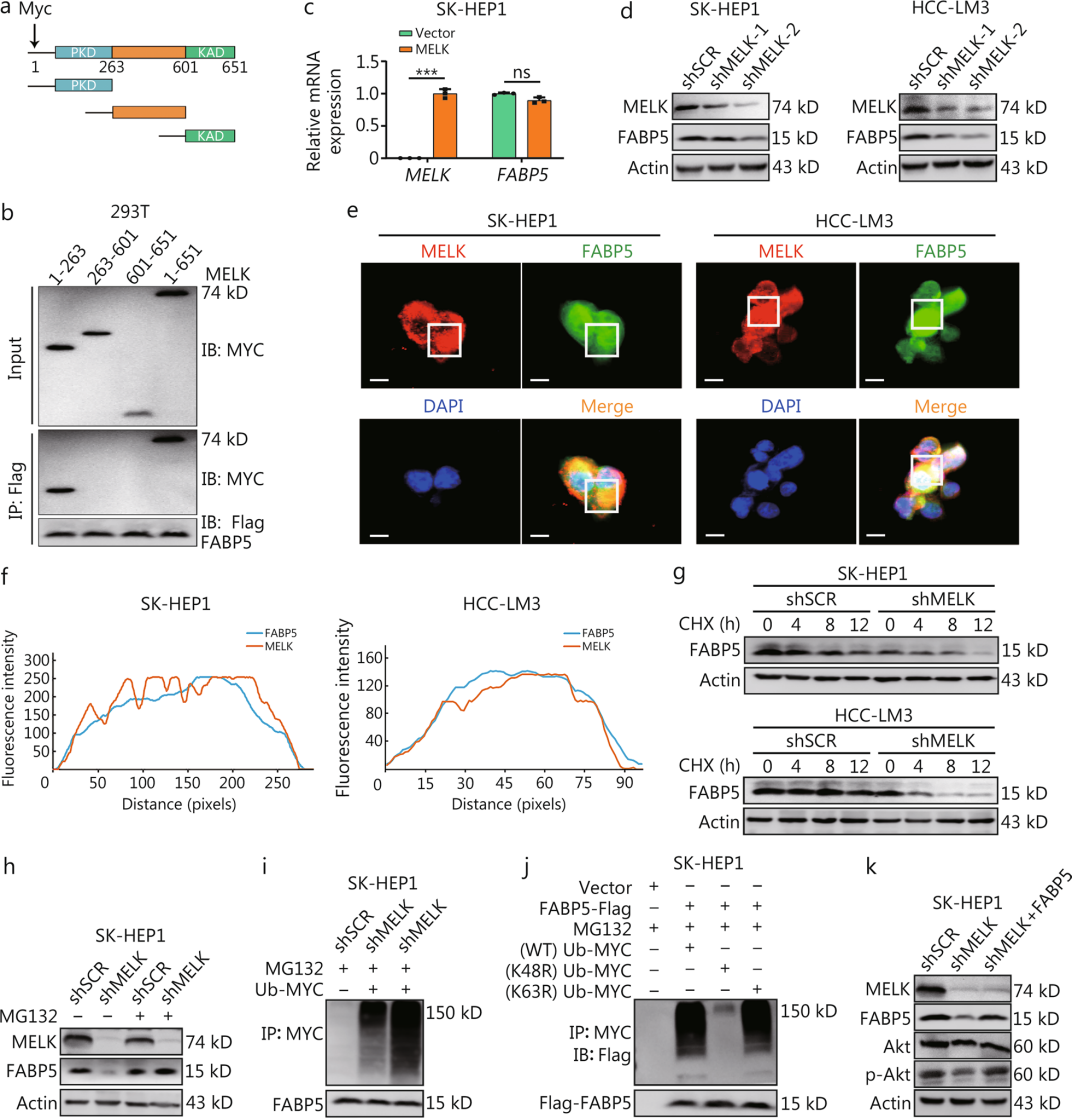
MELK decreased the Ub level of FABP5 to maintain its stability. **a** A schematic representation of the truncated structures of MELK proteins. **b** Western blotting shows the interaction between different truncated MELK and FABP5 proteins. **c** qPCR shows the relative mRNA expression of *MELK* and *FABP5* in SK-HEP1 cells. **d** Western blotting shows the expression of MELK and FABP5 in SK-HEP1 and HCC-LM3 cells. **e** Representative IF images show the interaction between MELK and FABP5 in SK-HEP1 and HCC-LM3 cells. **f** Colocalization analysis shows the interaction between FABP5 and MELK in SK-HEP1 and HCC-LM3 cells. **g** Western blotting shows FABP5 expression in SK-HEP1 and HCC-LM3 cells at different CHX treatment times. **h** Western blotting shows FABP5 expression in SK-HEP1 cells at MG132 treatment or not. **i** Western blotting shows Ub expression in SK-HEP1 cells with or without *MELK* knockdown after MG132 treatment. **j** Western blotting shows Ub-K48R and Ub-K63R levels in SK-HEP1 cells. **k** Western blotting shows MELK, FABP5, and Akt/p-Akt levels in SK-HEP1 hepatoma cells. Scale bar = 20 μm. ^***^P < 0.001, ns not significant. MELK maternal embryonic leucine zipper kinase, Ub ubiquitination, FABP5 fatty acid-binding protein 5, PKD protein kinase domain, KAD kinase-associated domain 1, MYC V-myc avian myelocytomatosis viral oncogene homolog, DAPI 4′,6-diamidino-2-phenylindole, CHX chlorhexidine, Akt protein kinase B, SCR scramble, MG132 Z-Leu-Leu-Leu-al

Since our findings showed that MELK binds to FABP5, we constructed three truncated MELK structures to detect the domain in MELK that interacts with FABP5. MELK contains a protein kinase domain (PKD; amino acids 1–263) and a kinase-associated domain (KAD; amino acids 601–651). Figure 5a shows a schematic diagram of the construction strategy. Our protein interaction experiments showed that the PKD of MELK binds to FABP5 (Fig. 5b). Interestingly, while FABP5 expression was unaffected when MELK was overexpressed in hepatoma SK-HEP1 and HCC-LM3 cells (Fig. 5c, Additional file 1: Fig. S8a), *MELK* knockdown significantly decreased FABP5 protein levels (Fig. 5d). We also observed that MELK colocalized with FABP5 in hepatoma SK-HEP1 and HCC-LM3 cells (Fig. 5e, f).

MELK does not affect the mRNA level but does affect the protein level of FABP5 in HCC cells. We constructed *MELK* knockdown cell lines to better understand the mechanism of how MELK affects FABP5 protein expression. When *MELK* was knocked down in hepatoma SK-HEP1 and HCC-LM3 cells, intracellular FABP5 content decreased with increasing chlorhexidine (CHX) exposure time after 0, 4, 8, and 12 h (Fig. 5g). Using ELISA, we also found that *MELK* knockdown reduced FABP5 secretion by hepatoma cells with CHX exposure time (Additional file 1: Fig. S8b). FABP5 protein expression was unaffected by *MELK* knockdown in hepatoma SK-HEP1 and HCC-LM3 cells after treatment with proteasome inhibitor MG132 (Fig. 5h, Additional file 1: Fig. S8c).

Ub is an important posttranslational modification for protein stability. When we overexpressed Ub molecules in hepatoma SK-HEP1 and HCC-LM3 cells, Ub molecules were more highly expressed in *MELK* knockdown cells (Fig. 5i, Additional file 1: Fig. S8d). Ub-K48 and Ub-K63 are the two most common polyubiquitinations that occur on lysine 48 (K48) or 63 (K63); K48 is key to affecting protein stability [33]. IP showed that Ub expression in hepatoma SK-HEP1 and HCC-LM3 cells was significantly inhibited with a K48R mutation in ubiquitin (Fig. 5j, Additional file 1: Fig. S8e). Therefore, we hypothesized that MELK influences the stability of FABP5 by regulating K48 Ub. *MELK* knockdown and FABP5 overexpression in hepatoma SK-HEP1 and HCC-LM3 cells showed that MELK inhibited the Ub-mediated degradation of FABP5, stabilizing FABP5 protein expression and thereby increasing the phosphorylation of downstream Akt (Fig. 5k, Additional file 1: Fig. S8f). Therefore, we hypothesized that MELK promotes PI3K/Akt/mTOR signaling by enhancing the protein stability of FABP5 in hepatoma cells.

### FABP5 is required for the antitumor effect of RFA and *MELK* knockdown in HCC

**Fig. 6:**
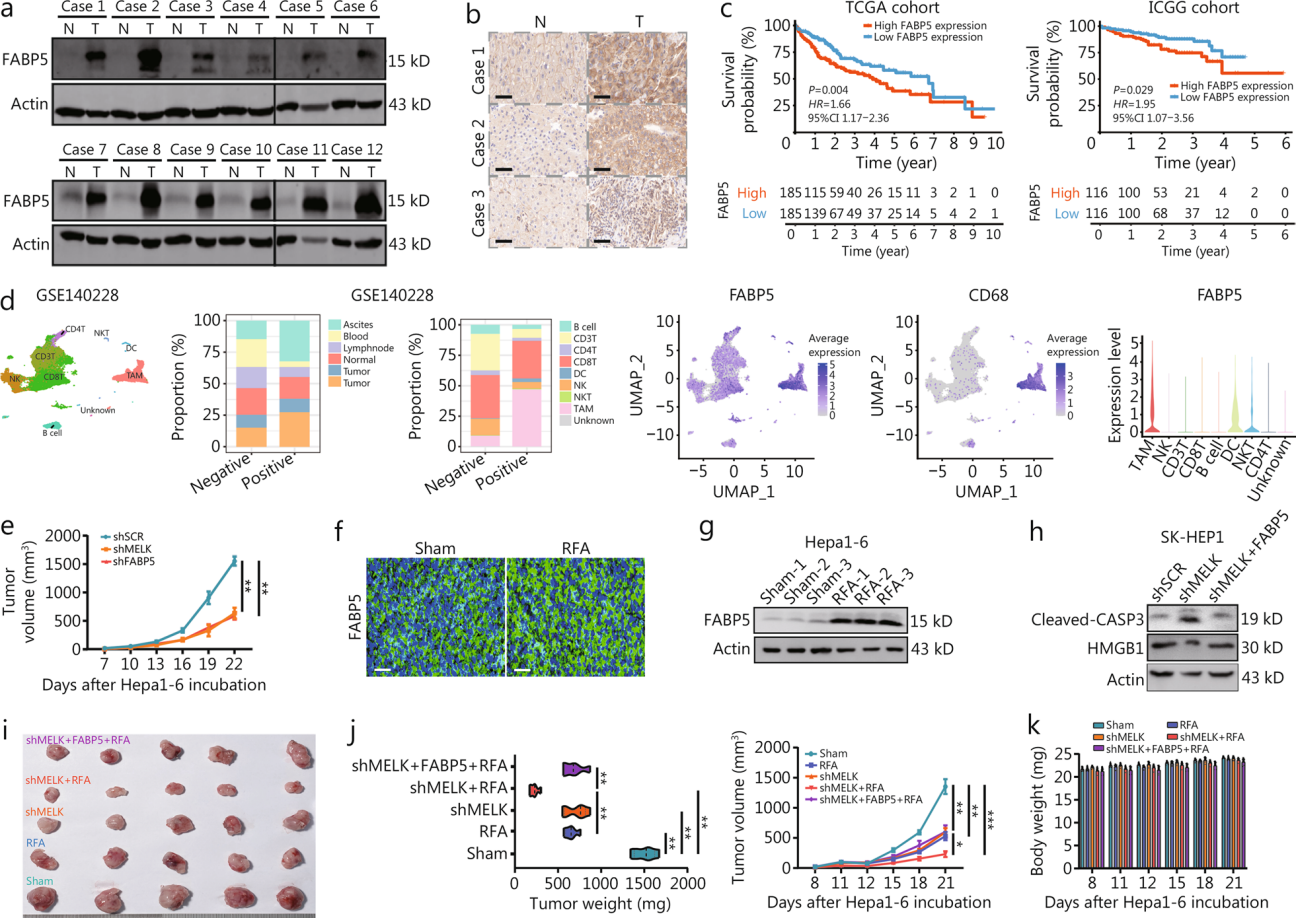
FABP5 is required for the antitumor effect of RFA treatment and *MELK* knockdown in HCC. **a** Western blotting shows the expression of FABP5 in the HCC and adjacent tissues of clinical samples. **b** Representative IHC images show FABP5 expression levels in the HCC and adjacent tissues of clinical samples. **c** The survival probability curves show the survival of patients with HCC and high or low FABP5 expression in the TCGA and ICGC databases.** d** Single-cell RNA sequencing analysis to identify the immune cell types infiltrating HCC. UMAP clustering of different clusters of immune cells and the FABP5-positive or negative percentage of positions or cell types. **e** The monitoring data shows Hepa1-6 subcutaneous tumor growth in the three groups. **f** Representative IF images show FABP5 expression in hepatoma cancer with sham or RFA treatment. The mouse model has been described and is same as in Fig. S1a. **g** Western blotting shows relative FABP5 expression in Hepa1-6 cells with sham or RFA treatment (*n* = 3). **h** Western blotting shows cleaved-CASP3, HMGB1, and actin levels in SK-HEP1 cells. **i** An image shows a Hepa1-6 subcutaneous transplantation tumor at day 21 (*n* = 5). **j** Tumor weights and volumes when the mice were euthanized. **k** A histogram of mouse body weights every 3 d until they were euthanized. Scale bar = 20 μm. ^*^P < 0.05, ^**^P < 0.01, ^***^P < 0.001. N normal, T tumor, FABP5 fatty acid-binding protein 5, MELK maternal embryonic leucine zipper kinase, RFA radiofrequency ablation, TCGA The Cancer Genome Atlas, ICGC International Cancer Genome Consortium, NKT natural killer T cell, NK natural killer cell, DC dendritic cell, TAM tumor-associated macrophage, UMAP uniform manifold approximation and projection, HMGB1 high mobility group box 1, IF immunofluorescence

We further explored the clinical relevance of FABP5 in HCC by analyzing two clinical liver cancer databases and examining clinical samples, which showed that the high expression of FABP5 in patients with liver cancer, compared with peritumoral areas (Fig. 6a, b, Additional file 1: Fig. S9a). The TCGA and ICGC databases suggested that high FABP5 expression was associated with poor prognosis in liver cancer (Fig. 6c), which was positively correlated with macrophage infiltration in HCC, especially M2 macrophages (Additional file 1: Fig. S9b).

Single-cell analysis of the GSE140228 cohort showed a higher FABP5 positive rate in the tumor edge and ascites, with TAMs the main cell clusters positive for FABP5 (Fig. 6d). Correlation and uniform manifold approximation and projection (UMAP) analyses suggested that FABP5 was highly correlated with macrophages and was significantly associated mainly with M2 macrophages (Fig. 6d). Another public dataset, GSE125449 was also evident that TAMs were the cell cluster with predominantly high FABP5 (Additional file 1: Fig. S9c). Moreover, single-cell analysis plots showed that TAMs were the cells with predominantly high FABP5 expression (Additional file 1: Fig. S9c).

In addition, we generated *MELK* knockout and *FABP5* knockout hepatoma cell lines for in-depth investigation (Additional file 1: Fig. S9d). The tumor inhibition effect of *FABP5* knockdown was found to be similar to that of *MELK* knockdown in the Hepa1-6 subcutaneous tumor bearing model (Fig. 6e, Additional file 1: Fig. S9e). Furthermore, deletion of *MELK* or *FABP5* was shown to inhibit TAM infiltration and promote CD8^+^ T cell infiltration, as demonstrated in Additional file 1: Fig. S9f.

Furthermore, FABP5 expression was significantly upregulated in the tumor tissue of the mouse hepatoma model following RFA treatment (Fig. 6f, g). Additionally, *MELK* knockdown in hepatoma cells appeared to induce hepatoma cell apoptosis and ICD, leading to inhibition of cell viability (Fig. 6h, Additional file 1: Fig. S9g, h). Conversely, FABP5 overexpression counteracted both of these patterns of cell death and the decrease in cell viability caused by *MELK* knockdown.

We constructed a Hepa1-6 tumor model to clarify further the role of FABP5 in regulating MELK-mediated RFA sensitivity in HCC. Tumor growth volume and weight were significantly smaller in the *MELK* knockdown and RFA-treated groups than those in control group. While the reductions in tumor volume and weight were greater in group combining *MELK* knockdown with RFA treatment, FABP5 overexpression prevented the enhanced effect of the combined treatment (Fig. 6i, j). Figure 6k indicates that there was little influence of mouse body weights in the 5 groups.

These results suggest that FABP5 is critical in regulating RFA efficacy and is required for the antitumor effect of RFA and *MELK* knockdown in HCC.

### Synthesis and validation of LNPs with arginylglycylaspartic acid (RGD)-MELK-siRNAs

Current MELK inhibitors mainly target its kinase activity but lack specific cell targeting. Using siRNA to target *MELK* mRNAs is the primary method to inhibit MELK expression. However, naked siRNAs are unstable and easily degraded by enzymes in the blood or engulfed by phagocytes in the organ. Therefore, it is difficult for them to reach the tumor site. LNPs are lipid-formed nanoparticles commonly used to deliver siRNAs and can enriched in tumor tissue through retention effects and active targeting. We added the RGD polypeptide to traditional LNPs to enhance targeting and permeability in hepatoma cells and exploit the pH-responsive signature to enable LNPs to precisely reach the tumor’s unique TME. Therefore, we encapsulated a MELK Cy5.5-conjugated siRNA in LNPs with a 100 nm diameter in a cationic-anionic binding manner and ligated the RGD polypeptide on the LNPs to target the liver cancer TME and subsequent tracing (Additional file 1: Fig. S10a), which we named RGD-LNP-siMELK-Cy5.5 (Nano-siMELK-Cy5.5). TEM showed the size and appearance of the Nano-siMELK-Cy5.5 LNPs (Additional file 1: Fig. S10b, c), suggesting that they were stable in quality, with a mostly 100 nm diameter.

We performed gel-delay assays to assess the binding force between siRNA and LNPs. siRNA-LNPs were prepared on the gel with different mass ratios. In Additional file 1: Fig. S10d, the disappearance of siRNA bands in the gel indicated that binding occurred between the LNPs and siRNAs, with siRNA binding observed at a mass ratio of 8:1, indicating that the siRNA was fully incorporated into the LNPs. The cumulative release of siRNAs into the environment was examined at pH 6.0 and 7.4 and was significantly higher at pH 6.0 than at pH 7.4 (Additional file 1: Fig. S10e). This finding suggests that the synthetic RGD LNPs provide pH-dependent siRNA release, with better release rates in the TME.

We also added equal volume Nano-siMELK-Cy5.5 to LO2 liver cells and HCC-LM3 liver cancer cells cultured in vitro. Liver cancer cells showed a consistently stronger ability to uptake LNPs than normal liver cells over time (Additional file 1: Fig. S10f). We detected *MELK* knockdown levels in SK-HEP1 and HCC-LM3 cells from protein levels to compare the direct addition of *MELK* siRNA (Naked-siMELK-Cy5.5) and Nano-siMELK-Cy5.5. Additional file 1: Fig. S10g shows that *MELK* knockdown efficiency was significantly higher with LNPs-siMELK (Nano-siMELK-Cy5.5) than with siMELK (Naked-siMELK-Cy5.5), with FABP5 inhibition also significantly increased. The CCK-8 assay results also suggested that while Naked-siMELK-Cy5.5 reduced hepatoma cell proliferation and viability, the effects were greater with Nano-siMELK-Cy5.5 (Additional file 1: Fig. S10h). The Cy5.5 signal in the serum supernatant further indicated that encapsulation in the LNPs made the siRNA difficult to eliminate, partly explaining the enhanced antitumor effect of the nanoliposome-encapsulated *MELK* siRNA (Additional file 1: Fig. S10i). We also compared the tumor targeting of RGD-LNPs and LNPs by thermal imaging. It was evident that the RGD polypeptide significantly increased the targeting of LNPs to tumors (Additional file 1: Fig. S10j). We verified that the synthesized Nano-siMELK-Cy5.5 was of good quality and showed good targeting to hepatoma cells and tissues using TEM and confocal laser scanning microscopy in vitro and in vivo. Its MELK interference efficiency was as expected, indicating it is suitable for subsequent use.

### LNPs targeting tumor cell-intrinsic MELK enhance RFA-induced antitumor immune effects in HCC

**Fig. 7:**
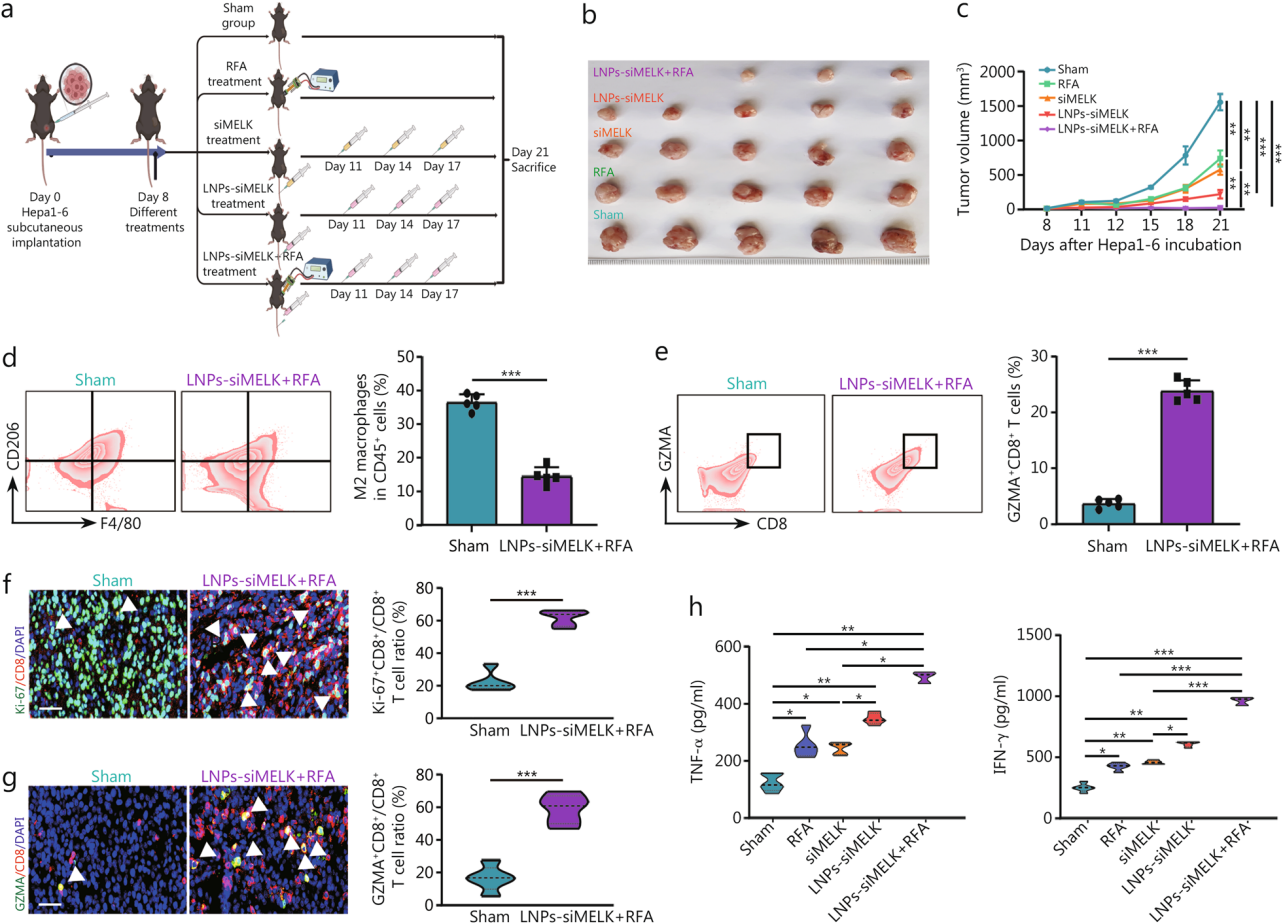
LNPs-siRNAs targeting tumor cell-intrinsic MELK enhance RFA-induced antitumor immune effects in HCC. **a** An animal model illustration shows the different treatments in the 5 groups. **b** An image of Hepa1-6 tumor tissue isolated when the mouse was euthanized (*n* = 5). **c** The monitoring data shows Hepa1-6 subcutaneous tumor growth in the 5 groups. Flow cytometry analysis and percentages of M2 macrophages (**d**) and GZMA^+^CD8^+^ T cells (**e**) in Hepa1-6 tumor tissue (*n* = 5). **f** IF images show the Ki-67 and CD8 in Hepa1-6 tumor tissues. Proportions of Ki-67^+^CD8^+^ T cells among CD8^+^ T cells. White arrows indicate CD8^+^ cells expressing Ki-67. **g** The IF images show GZMA and CD8 in Hepa1-6 tumor tissues. Proportions of GZMA^+^CD8^+^ T cells among CD8^+^ T cells. White arrows indicate CD8^+^ cells expressing GZMA. **h** ELISA shows the TNF-α and INF-γ concentration in Hepa1-6 tumor tissues from the five groups. Scale bar = 20 μm. ^*^P < 0.05, ^**^P < 0.01, ^***^P < 0.001. RFA radiofrequency ablation, LNP lipid nanoparticle, MELK maternal embryonic leucine zipper kinase, GZMA granzyme A, TNF-α tumor necrosis factor-α, INF-γ interferon-γ, DAPI 4′,6-diamidino-2-phenylindole

Our previous results showed that *MELK* knockdown in liver cancer cells could enhance RFA-induced antitumor effects. We similarly found that LNPs containing siMELK (Nano-siMELK-Cy5.5, LNPs-siMELK) inhibited liver cancer cell viability and enhanced tumor cell inhibition under the exogenous application of 47 °C (Additional file 1: Fig. S11a, b). We treated mice with siMELK or LNPs-siMELK injected directly into their tail vein every 3 d in groups with or without RFA treatment and sacrificed the mice to harvest tumor tissues on day 21 and also confirmed its efficiency (Fig. 7a). Both RFA or siMELK alone inhibited tumor growth to some extent, including tumor volume, and progression rate. The LNPs-siMELK had a greater tumor-suppressive effect than the siMELK, especially when combined with RFA, significantly increasing its inhibitory effect (Fig. 7b, c, Additional file 1: Fig. S11c).

**Fig. 8:**
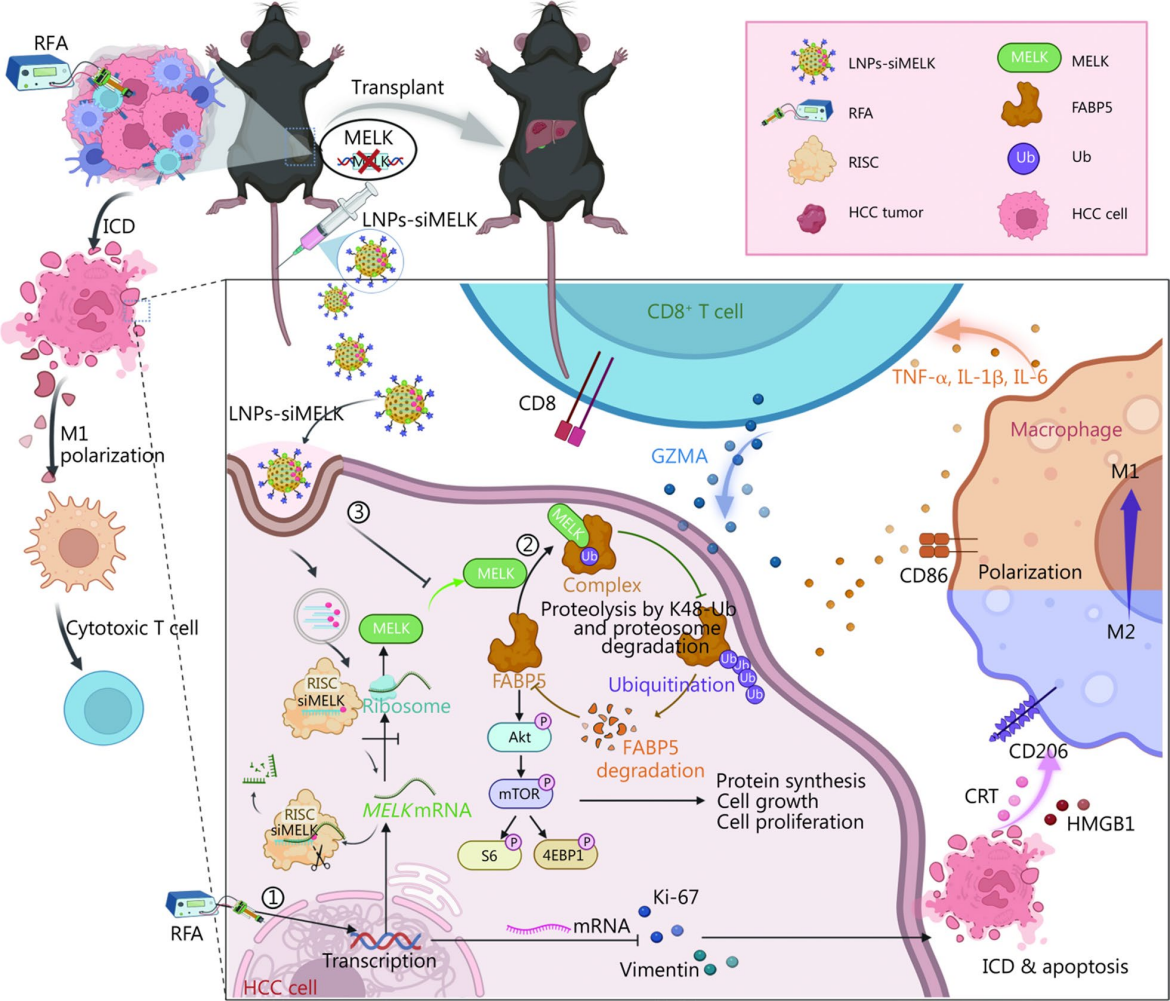
The molecular mechanism flowchart shows that MELK stabilizes FABP5 to amplify the anti-hepatoma cancer effect of RFA. ① RFA can promote immunogenic cell death and immune activation in hepatocellular carcinoma. ② MELK promotes ubiquitination degradation of FABP5, thereby inhibiting downstream AKT/mTOR signals and promoting the malignant progression of tumors. ③ The LNP platform can promote the antitumor effect of RFA, mainly by promoting ICD and immune activation in HCC. MELK maternal embryonic leucine zipper kinase, RFA radiofrequency ablation, LNP lipid nanoparticle, ICD immunogenic cell death, RISC RNA-induced silencing complex, HCC hepatocellular carcinoma, Ub ubiquitination, FABP5 fatty acid-binding protein 5, GZMA granzyme A, Akt protein kinase B, mTOR mammalian target of rapamycin, S6 ribosomal protein S6, 4EBP1 eukaryotic translation initiation factor 4E binding protein 1, TNF-α tumor necrosis factor-α, IL-1β interleukin-1β, IL-6 interleukin-6, CRT calreticulin, HMGB1 high mobility group box 1

This study provides a new practical approach to clinically target MELK, which will be beneficial in developing clinical treatments for liver cancer. When flow cytometry was used to detect the proportion of immune cells in tumor tissues, the proportions of TAMs were significantly lower, M1 macrophages were higher (Additional file 1: Fig. S11d, e), and M2 macrophages (Fig. 7d) were lower in group co-treated with LNPs-siMELK and RFA than those in sham group. Besides, the proportions of granzyme A (GZMA)^+^CD8^+^ T cells representing immune activation were significantly higher than in sham group (Fig. 7e). We also labeled CD8^+^ T cells in tumor tissues by the double-labeling IF method and labeled proliferative CD8^+^ T cells (Fig. 7f, g). While CD8^+^ T cells were the main proliferating cells in the group co-treated with LNPs-siMELK and RFA, they showed little proliferation in the sham group (Fig. 7f). After co-labeling with GZMA, it was evident that CD8^+^ T cells with killing function were significantly more numerous in the group co-treated with LNPs-siMELK and RFA than in the sham group (Fig. 7g). We then quantified the levels of cytokines related to immune activation by ELISA, including TNF-α, interferon-γ (INF-γ), IL-2, and IL-12. While both RFA and siMELK alone could promote TNF-α, INF-γ, IL-2, and IL-12 secretion to some extent, the effect was greater with LNPs-siMELK, especially when combined with RFA, significantly increasing TNF-α, INF-γ, IL-2, and IL-12 in the TME (Fig. 7h, Additional file 1: Fig. S12a). Importantly, the weight records of mice and the staining results of important organs showed that our treatment did not harm their health, and its biosafety was high (Additional file 1: Fig. S12b, c). The molecular mechanism flowchart showed that MELK stabilizes FABP5 to amplify the anti-hepatoma cancer effect of RFA (Fig. 8).

## Discussion

RFA is currently the most important ablation technique in treating liver cancer. RFA is the first-line treatment for patients with inoperable liver cancer due to its operability, high safety, effectiveness, high organic substance preservation rate, and high repeatability [34]. RFA electrodes inside the tumor can cause coagulation necrosis after increasing the temperature through high-frequency AC electricity and can also kill the tumor by regulating its immune microenvironment [35]. In addition to its regulatory effect on CD8^+^ T cells, our results showed that RFA of HCC is closely related to TAM infiltration and polarization. RFA induced the M1 polarization of HCC-associated macrophages but inhibited their M2 polarization. These results provide a basis for targeting the HCC immune microenvironment to promote the sensitivity of HCC RFA therapy. However, due to the spatial limitation of RFA, the “thermal precipitation effect” and the short duration of the activated immune response in the indirect tumor-killing effect are the key factors limiting the efficacy of RFA, leading to the recurrence and progression of residual cancer after RFA.

Our study found that MELK expression was elevated after RFA treatment. It was further confirmed by clinical cohort analysis that increased MELK expression in the tissues of patients with early HCC recurrence was strongly associated with poorer overall survival and disease-free survival. Both in vivo and in vitro experiments suggested that *MELK* knockdown inhibited hepatoma cell growth, invasion, stemness, and tumorigenesis by inducing apoptosis and ICD. Our results also confirmed that *MELK* is a potential oncogene in liver cancer. *MELK* knockdown inhibited liver cancer cell proliferation, migration, tumor growth, and lung metastasis. Importantly, combining *MELK* knockdown with RFA could directly kill tumor cells and inhibit liver cancer growth by enhancing RFA-induced apoptosis. This finding also confirmed that MELK could affect the sensitivity of liver cancer to RFA by regulating tumor cell growth and survival.

Previous studies have found that MELK expression is closely associated with immune cell infiltration, including TAM polarization [12, 36]. The most abundant group of inflammatory cells in the TME, TAMs play a leading role as the coordinator of cancer-related inflammation [37]. They exert antitumor and antitumor effects in the TME through their M1 and M2 polarization, respectively [38]. Therefore, targeting macrophage polarization is an effective strategy to combat tumor progression. However, the role of macrophage polarization in regulating the efficacy of RFA treatment must be further examined. This study confirmed that *MELK* knockdown in HCC cells promoted the M1 polarization of macrophages but inhibited the infiltration of HCC TAMs and the M2 polarization of macrophages. MELK-mediated macrophage polarization also indirectly affected the recruitment and activity of CD8^+^ T cells, further inhibiting tumor growth. Importantly, TAM infiltration and polarization also play a key role in the RFA-induced changes in the immune microenvironment of liver cancer. Therefore, combining *MELK* knockdown with RFA treatment could further inhibit HCC TAM infiltration and the M2 polarization of macrophages, and promote the M1 polarization of macrophages. Changes in TAM polarization also indirectly affect the recruitment of tumor-killing CD8^+^ T cells to exert antitumor effects. This finding suggests that MELK is an important regulatory molecule whose knockdown increases the RFA-induced immune effect on liver cancer. Targeting MELK may be an effective strategy to enhance the sensitivity of liver cancer to RFA and exert antitumor effects by amplifying and prolonging the RFA-induced immune response against tumor progression.

The fatty acid transporter FABP5 is expressed in liver, small intestine, and kidney tissue. FABP5 is essential in the progression of cancer and metabolic diseases [39]. Some studies have found that the FABP5/hypoxia-inducible factor 1 subunit alpha axis promotes HCC cell proliferation, and its expression is associated with poor prognosis [31, 40, 41]. Our study found that FABP5 expression was elevated in liver cancer tissues and HCC-animal models after RFA treatment, suggesting that FABP5 is associated with poor liver cancer prognosis. In addition, FABP5 could promote tumor progression by activating PI3K/Akt/mTOR signaling [42]. Regarding molecular mechanism, our study found that MELK inhibits FABP5 Ub and stabilizes FABP5 protein levels through its interaction with FABP5, thereby promoting PI3K/Akt/mTOR signaling. The overactivation of the PI3K/Akt/mTOR signaling axis is closely associated with tumor cell proliferation, growth, death, and other processes. We found that the tumor cell death and antitumor effects caused by *MELK* knockdown combined with liver cancer thermal ablation may be closely related to FABP5 expression. This finding also partially elucidates the regulatory mechanism of *MELK* knockdown in enhancing RFA-induced tumor killing. In addition, we found that high FABP5 expression was closely involved in immune cell infiltration, including by TAMs. FABP5 is required for ICD induced by RFA and *MELK* knockdown, which activates the antitumor immune response in HCC. In addition, tumor-derived MELK expression promotes liver cancer progression by regulating macrophage polarization and CD8^+^ T cell infiltration. Therefore, considering the antitumor properties of MELK in liver cancer, this study provides strong evidence to support the powerful antitumor potential of targeting MELK in combination with RFA in liver cancer.

Although our findings greatly encourage the hope of treating patients with liver cancer, we still face unknown difficulties. Due to tumor heterogeneity, whether all patients respond well to *MELK* knockdowns remains a concern. The level of MELK expression in tumor tissues of patients with liver cancer varies from individual to individual, and it is still a question worth exploring whether all patients are effective in combination therapy.

## Conclusion

Altogether, these findings suggest that high TAM invasion and high MELK expression promote HCC recurrence after RFA treatment. Therefore, we have designed a viable therapeutic nanomedical drug that encapsulates a pH-responsive lipid nanoshell with siMELK (LNPs-siMELK) for radiosensitization. It is based on enhancing the RFA-induced tumor-killing effect and promoting the remodeling of the tumor immune microenvironment for the sustained effectiveness of RFA therapy, including TAM M1 polarization and CD8^+^ T cell recruitment to kill tumors. Animal experiments suggest that LNPs-siMELK has higher tumor targeting and longer efficacy than Naked-siMELK and has a greater effect when combined with RFA. Our data provide favorable evidence for exploring the direct killing effect of RFA enhanced by *MELK* knockdown and the associated indirect killing effect via the immune response and provide a new theoretical basis for precision medicine.

## Supplementary Information

**Additional file 1**: **Materials and methods**. **Table S1** Antibodies for Western blotting, IF, IHC, and IP. **Table S2** FACS antibodies. **Table S3** shRNA and siRNA sequences. **Table S4** Primers for qPCR. **Table S5** Clinical arrays. **Fig. S1** RFA inhibits tumor progression and induces immune cell infiltration. **Fig. S2** RFA treatment increased MELK expression, an independent risk factor in patients with HCC. **Fig. S3** *MELK* knockdown enhances HCC sensitivity to heat treatment and RFA efficacy. **Fig. S4** *MELK* knockdown promotes RFA-induced apoptosis and immunogenic death in hepatoma cells. **Fig. S5** *MELK* knockdown enhances RFA-induced antitumor immune effects in HCC. **Fig. S6** The changes in immune cell infiltration in liver tumors after RFA treatment or *MELK* knockdown. **Fig. S7** Tumor cell-intrinsic MELK enhanced the PI3K/Akt/mTOR signal axis and FABP5 interaction. **Fig. S8** *MELK* decreased the Ub level of FABP5 to maintain its stability. **Fig. S9** FABP5 is required for the antitumor effect of RFA treatment and *MELK* knockdown in HCC. **Fig. S10** LNPs with RGD-MELK-siRNAs were synthesized. **Fig. S11** LNPs-siRNAs targeting tumor cell-intrinsic MELK enhance RFA-induced antitumor immune effects in HCC. **Fig. S12** The immune effect and therapeutic toxicity detection.

## Data Availability

Data is openly available in a public repository.

## Abbreviations

Akt: Protein kinase B
BCL2: B-cell leukemia/lymphoma 2
CASP3: Caspase-3
cDAMP: Compositive-damage-associated molecular patterns
CXCL10: C-X-C motif chemokine ligand 10
Cy5.5: Cyanine 5.5
CCL2: C–C motif chemokine ligand 2
CHX: Chlorhexidine
DAPI: 4′,6-Diamidino-2-phenylindole
DEG: Differentially expressed gene
4EBP1: Eukaryotic translation initiation factor 4E binding protein 1
FABP5: Fatty acid-binding protein 5
FC: Fold change
GSEA: Gene set enrichment analysis
GO: Gene Ontology
HCC: Hepatocellular carcinoma
HMGB1: High mobility group box 1
ICD: Immunogenic cell death
IL-6: Interleukin-6
ICGC: International cancer genome consortium
IF: Immunofluorescence
IHC: Immunohistochemistry
INF-γ: Interferon-γ
KAD: Kinase-associated domain 1
KEGG: Kyoto encyclopedia of genes and genomes
LIHC: TCGA-liver HCC
LNPs: Lipid nanoparticles
mTOR: Mammalian target of rapamycin
MELK: Maternal embryonic leucine zipper kinase
MPK38: Murine protein serine-threonine kinase 38
PKD: Protein kinase domain
RARP: Poly (ADP-ribose) polymerase
PI3K: Phosphoinositide 3-kinase
RFA: Radiofrequency ablation
RGD: Arginylglycylaspartic acid
siRNA: Small interfering RNA
shRNA: Small hairpin RNA
S6: Ribosomal protein S6
TME: Tumor microenvironment
TCGA: The cancer genome atlas
TEM: Transmission electron microscope
TNF-α: Tumor necrosis factor-α
UMAP: Uniform manifold approximation and projection
Ub: Ubiquitination
