# Supplementary material for: MELK prevents radiofrequency ablation-induced immunogenic cell death and antitumor immune response by stabilizing FABP5 in hepatocellular malignancies
Source: Mil Med Res. 2025 Jan 27;12:5. doi: 10.1186/s40779-024-00588-7 (PMC11773770; doi:10.1186/s40779-024-00588-7)
Supplement: Supplementary file 1 — Additional file 1: Materials and methods. Table S1 Antibodies for Western blotting, IF, IHC, and IP. Table S2 FACS antibodies. Table S3 shRNA and siRNA sequences. Table S4 Primers for qPCR. Table S5 Clinical arrays. Fig. S1 RFA inhibits tumor progression and induces immune cell infiltration. Fig. S2 RFA treatment increased MELK expression, an independent risk factor in patients with HCC. Fig. S3 MELK knockdown enhances HCC sensitivity to heat treatment and RFA efficacy. Fig. S4 MELK knockdown promotes RFA-induced apoptosis and immunogenic death in hepatoma cells. Fig. S5 MELK knockdown enhances RFA-induced antitumor immune effects in HCC. Fig. S6 The changes in immune cell infiltration in liver tumors after RFA treatment or MELK knockdown. Fig. S7 Tumor cell-intrinsic MELK enhanced the PI3K/Akt/mTOR signal axis and FABP5 interaction. Fig. S8 MELK decreased the Ub level of FABP5 to maintain its stability. Fig. S9 FABP5 is required for the antitumor effect of RFA treatment and MELK knockdown in HCC. Fig. S10 LNPs with RGD-MELK-siRNAs were synthesized. Fig. S11 LNPs-siRNAs targeting tumor cell-intrinsic MELK enhance RFA-induced antitumor immune effects in HCC. Fig. S12 The immune effect and therapeutic toxicity detection. [file 40779_2024_588_MOESM1_ESM.pdf]

Materials and methods

Plasmid transfection

Lipo2000 (Thermo Fisher Scientific, USA) was applied for plasmid transfection. The target plasmid and 2-times the volume of Lipo2000 were mixed and incubated at room temperature for 20 min before being added to the cells. Removed it after 6 – 8 h of culture, and RNA and protein were extracted after 48 h of continuous culturing. Exogenously overexpressed MELK and FABP5 proteins in 293T cell lines and subsequent protein interaction tests were performed.

Construction and screening of stable cell lines

The target cells were infected 24 h later by adding the appropriate amount of virus for the cells' multiplicity of infection value and the virus concentration. Then, puromycin was added to screen out stable cell lines, and cell RNA and protein were extracted to detect knockdown efficiency. Hepa1-6-luciferase cell lines with the stable expression of luciferase were constructed to retain its primary properties with promising bioluminescence imaging ability.

5-Ethynyl-2-deoxyuridine (EdU)-based cell proliferation assay

Dulbecco's modified Eagle's medium (DMEM) complete medium containing 10 μmol/L of the EdU probe (Beyotime Biotechnology, China) was added for 3 – 5 h. Next, a 4% paraformaldehyde (PFA) solution was added to fix it. Next, the click additive solution was dissolved in deionized water, and 500 μl was added in the dark for 30 min. Then, DAPI was added to detect nuclear DNA. After the fluorescent anti-quenching slides were sealed, fluorescence microscopy was used to observe the EdU-stained cells.

Live/dead cell staining assay

Calcein AM and propidium iodide (PI) were added to cells for 10 – 15 min, then observed under a fluorescence microscope.

Western blotting

The tissue or cell samples of liver cancer were lysed at 4 °C in RIPA solution with a mixture of the Protease Inhibitor Cocktail and PhosSTOP (inhibitor tablets for phosphatase). Protein samples were separated by sodium dodecyl sulfate-polyacrylamide gel electrophoresis and transferred onto polyvinylidene fluoride membranes. Next, the corresponding antibodies were incubated. Finally, the corresponding bands were developed using the enhanced chemiluminescence substrate by a chemical method. The antibodies used in this study are listed in the **Additional file 1: Table S1**.

Co-immunoprecipitation (Co-IP)

Co-IP is an experimental method for detecting protein-protein interactions. Cell samples were lysed the same as the Western blotting method. Antibodies were adsorbed onto beads. Next, mix beads and lysate at

4 °C overnight on a 360° shaker. Then, subject to the Western blotting process described above.

RNA extraction and quantitative real-time PCR (qPCR)

RNA was extracted and reverse-transcribed using the RNA Rapid Extraction Kit (EZBioscience, USA) and a commercial kit (Thermo Fisher Scientific, USA) according to the manufacturer's recommended protocol. SYBR-Green fluorescent probe and real-time fluorescence qPCR were performed using a Bio-Rad qPCR instrument (Bio-Rad Laboratories, USA), followed by data analysis. The primers used in this study are provided in the **Additional file 1: Table S4**.

Cell proliferation/toxicity

First, the cells were gently blown until a single-cell granular suspension formed. Then, the suspension was diluted to 25 cells/μl based on the cell count results, and 200 μl of the cell suspension was added to each well of 96-well plates (5000 cells/well). After the cells were cultured to a good-adherent condition, the DMEM complete culture medium was replaced with DMEM complete culture medium containing 10% Cell Counting Kit-8 (CCK-8; Beyotime Biotechnology, China) solution and cultured for 2 – 4 h. Then, the cells were removed and placed into a microplate reader. The oscillation time of the microplate reader was set to 30 s, and absorbance was detected at 450 nm.

Tissue fixation, paraffin-embedding, and sectioning

After the mice were sacrificed using CO_2, HCC tumor tissue samples were quickly separated, washed 3 times with PBS, and fixed at room temperature with 4% PFA for 2 – 7 d. The tissue was made transparent by dehydration with an ethanol gradient and xylene treatment before paraffin embedding. The paraffin-embedded tissue was sectioned into 4 μm thick slices and placed into an oven at 65 °C overnight for subsequent IHC/IF dyeing.

Enzyme-linked immunosorbent assays (ELISA) & multiplex cytokine array assay

Tumor necrosis factor (TNF)-α and interferon (INF)-γ levels in the tissue lysates were measured using relative ELISA Kit (Invitrogen/Thermo Fisher Scientific, USA), according to the manufacturer's instructions. Cytokine array kits were purchased from R&D Systems (USA). Multiplex cytokine assay is a membrane-based sandwich immunoassay.

RNA sequencing differential analysis

Liver cancer tissues from C57BL/6 mice and hepatoma cell lines with different treatments were collected and then processed following the 10× Genomics protocol. DEGs in the heatmap were identified between the different tumor microenvironments (TMEs). The raw sequencing data were analyzed through the Cell Ranger pipeline, and the relative Cell Ranger R package was used to perform downstream RNA-sequencing analysis. Benjamini-Hochberg's method was adjusted in the *P*-values of multiple tests, and the false

discovery rate (FDR) < 0.01 and $|\log_2$ fold change (FC)$| \geq 1$ was applied as the significance level threshold.

Liquid chromatography-mass spectrometry (LC-MS)

SK-HEP1 cells expressing MELK were lysed in IP buffer (20 mmol/L Tris-HCl, pH 8.0, 150 mmol/L NaCl, 2 mmol/L EDTA, and 1% Nonidet P-40) containing a protease inhibitor cocktail (Roche, Swiss). The lysates were then added to beads that already absorbed the MELK antibody, then incubated together at 4 °C overnight. The collected beads as it described in the Co-IP part. Fractions of the bed volume were collected and resolved through SDS-PAGE and silver staining. LC-MS/MS sequencing was then carried out for the gel bands by Qinglian Bio (Beijing, China).

Open database

The clinical HCC relative databases were downloaded from previously published public Gene Expression Omnibus (GEO) data base, The Cancer Genome Atlas (TCGA), and International Cancer Genome Consortium (ICGC) data centers. We further analyzed the genes closely related to this research.

CIBERSORT analyses

CIBERSORT is an open data analysis tool, we use its website (https://cibersortx.stanford.edu/) to analyze the immune cell types infiltration in HCC tissue. CIBERSORT outputs the proportion of immune cell infiltration based on the reference data set, the sum of which is 1 for all cell types.

Extraction and analysis of clinical data

The study selection was performed by two independent investigators (Bu-Fu Tang and Wang-Ting Xu). Studies that analyzed risk factors and prognosis models among patients with HCC. Raw data were extracted using a standard form, which included 75 HCC samples and the matched 64 para-HCC samples from patients diagnosed with primary HCC and underwent resection at the Lishui Hospital, School of Medicine, Zhejiang University between 2009 and 2016 with corresponding clinical information. Such as age, gender, pathological diagnosis, TNM stage, clinical diagnosis, tumor size, recurrence, metastasis, clinical tumor-related indicators, treatment, outcome, survival time, and patient characteristics shown in **Additional file 1: Table S5**. The study was approved by the Human Ethics Committee of Lishui Central Hospital [(2024) No. 177] by the ethical guidelines for medical and biological research involving human subjects (2016), the World Medical Association Declaration of Helsinki and the CIOMS International Ethical Guidelines for Health-Related Research Involving Humans. The following are the key inclusion criteria: HCC patients with primary HCC (cholangiocarcinoma or mixed liver cancer) only, aged 18 – 75 years, without distant metastases. The key exclusion criteria are as follows: 1) primary HCC patients who died from other causes, and 2) primary HCC patients with other primary cancers.

Small animal in vivo imaging

Hepa1-6-luciferase cell lines in vivo imaging of small animals after successful tumor bearing. Photon Imager Optima In Vivo Imaging System (IVIS) for small animals (Biospace Lab, France) was applied to imaging tumor tissue in C57BL/6J male mice aged 6 – 8 weeks in this study. After intraperitoneally injecting the substrate D-luciferin potassium salt (Beyotime, China, 10 μl/g body weight) for 10 min, the fluorescence signal reached the strongest stable plateau, and then imaging analysis was performed with a small animal imaging instrument. The images obtained were processed using M3VISION in vivo imaging analysis software.

Table S1 Antibodies for Western blotting, IF, IHC, and IP

Name	Dilution	Species	Manufacture	Cat No.	Application
MELK	1:1000<br>1:200<br>—	Rabbit	Abcam	ab273015<br>ab245574	Western blotting<br>IF, IHC<br>IP
FABP5	1:1000<br>1:200<br>—	Rabbit	Abcam	ab255276<br>ab84028	Western blotting<br>IF, IHC<br>IP
Phosphor-STAT3 (S727)	1:500	Rabbit	Abcam	ab32143	IHC
CD8A	1:100	Rat	Abcam	ab308264	IF
HMGB1	1:1000	Rabbit	Abcam	ab18256	Western blotting
CD31	1:800	Rabbit	Cell Signaling Technology	#3528	IF
Vimentin	1:200	Rabbit	Cell Signaling Technology	#5741	IHC
PCNA	1:400	Rabbit	Cell Signaling Technology	#13110	IF
Calreticulin (CRT)	1:1000<br>1:400	Rabbit	Cell Signaling Technology	#12238	Western blotting<br>IF
Ki-67	1:400	Rabbit	Cell Signaling Technology	#9027	IHC, IF
CD86	1:100	Rabbit	Abcam	ab239075	IF
Cleaved PARP	1:1000	Rabbit	Cell Signaling Technology	#9541	Western blotting
Cleaved Caspase-3	1:1000<br>1:400	Rabbit	Cell Signaling Technology	#9661	Western blotting<br>IHC
BCL-2	1:1000	Rabbit	Cell Signaling Technology	#3498	Western blotting
F4/80	1:400	Rabbit	Cell Signaling Technology	#30325	IF
CD206	1:400	Rabbit	Cell Signaling Technology	#24595	IF
GZMA	1:100	Rabbit	Invitrogen	PA5-96539	IF
mTOR	1:1000	Rabbit	Cell Signaling Technology	#2983	Western blotting
p-mTOR	1:1000	Rabbit	Cell Signaling Technology	#5536	Western blotting
S6K	1:1000	Rabbit	Abcam	ab32529	Western blotting
p-S6K	1:1000	Rabbit	Abcam	ab131436	Western blotting
4EBP1	1:1000	Rabbit	Cell Signaling Technology	#9452	Western blotting
p-4EBP1	1:1000	Rabbit	Cell Signaling Technology	#2855	Western blotting
Myc	1:1000<br>—	Rabbit	Cell Signaling Technology	#9402	Western blotting<br>IP

Flag	1:1000<br>—	Rabbit	Cell Signaling Technology	#14793	Western blotting<br>IP
p-Akt	1:1000	Rabbit	Cell Signaling Technology	#4060	Western blotting
Akt	1:1000	Rabbit	Cell Signaling Technology	#4691	Western blotting
Actin	1:4000	Mouse	Abcam	ab8226	Western blotting
Goat anti-rabbit IgG-HRP	1:10,000	Goat	ABclonal Technology	AS014	Western blotting
Goat anti-mouse IgG-HRP	1:10,000	Goat	ABclonal Technology	AS003	Western blotting

MELK maternal embryonic leucine zipper kinase, *FABP5* fatty acid binding protein 5, *STAT3* signal transducer and activator of transcription 3, *IF* immunofluorescence, *IHC* immunohistochemistry, *IP* immunoprecipitation, *HMGB1* high mobility group box 1, *PCNA* proliferating cell nuclear antigen, *PARP* poly ADP-ribose polymerase, *BCL-2* B-cell lymphoma-2, *GZMA* granzyme A, *mTOR* mammalian target of rapamycin, *S6K* S6 kinases, *4EBP1* eukaryotic translation initiation factor 4E (eIF4E)-binding protein 1, *Myc* V-myc avian myelocytomatosis viral oncogene homolog, *Akt* protein kinase B, *HRP* horseradish peroxidase, — no data

Table S2 FACS antibodies

Name	Fluorophore	Clone	Manufacture	Cat No.
F4/80	PE	BM8	Biolegend	123110
CD206	FITC	C068C2	Biolegend	141703
CD45	APC	30-F11	Biolegend	103112
CD11b	FITC	M1/70	Biolegend	101205
CD16/32	FITC	93	Biolegend	101306
CD8	FITC	5H10-1	Biolegend	100803
GZMA	PE	3G8.5	Biolegend	149703

FACS fluorescence-activated cell sorting, *GZMA* granzyme A, *PE* R-phycoerythrin, *FITC* fluorescein Isothiocyanate, *APC* allophycocyanin

Table S3 shRNA and siRNA sequences

Name	Sequences
H-shMELK-1	5′-CAGAAACAACAGGCAAACAAT-3′
H-shMELK-2	5′-GCCTGAAAGAAACTCCAATTA-3′
M-shMELK-1	5′-GCAGCTCCTGAACTAATACAA-3′
M-shMELK-2	5′-GCTGGATTGATAGACTATGAA-3′
siMELK-1	5′-GCATTCTGCTTCTTCAACA-3′
siMELK-2	5′-CCAAAGACUCCAGUUAAUA-3′

shRNA small hairpin RNA, *MELK* maternal embryonic leucine zipper kinase

Table S4 Primers for qPCR

Name	Sequences	Supplier
h-actin	Forward: CATGTACGTTGCTATCCAGGC	TSINGKE Biological Technology, Beijing, China
	Reverse: CTCCTTAATGTCACGCACGAT	
h-MELK	Forward: TCTCCCAGTAGCATTCTGCTT	
	Reverse: TGATCCAGGGATGGTTCAATAGA	
h-FABP5	Forward: TGAAGGAGCTAGGAGTGGGAA	
	Reverse: TGCACCATCTGTAAAGTTGCAG	

qPCR quantitative real-time PCR, *MELK* maternal embryonic leucine zipper kinase, *FABP5* fatty acid binding protein 5

Table S5 Clinical arrays

ID	Futime	Fustat	Age (years)	Gender	Grade	TNM	TNM stage	AFP (ng/ml)	Recurrence	MELK	Type
P1	620	0	< 60	Male	2	T3N0M0	III + IV	≥ 400	Yes	30.5700	Low
P2	434	1	< 60	Male	2	T3N0M0	III + IV	≥ 400	Yes	88.9312	High
P3	1231	1	< 60	Male	2	T3N0M0	III + IV	≥ 400	No	80.8745	High
P4	404	0	< 60	Female	2	T1bN0M0	I + II	< 400	Yes	41.9452	Low
P5	403	1	< 60	Female	2	T3N0M0	III + IV	< 400	Yes	92.9011	High
P6	742	1	< 60	Male	3	T2N0M0	I + II	≥ 400	Yes	80.6294	High
P7	380	1	< 60	Male	2	T2N0M0	I + II	< 400	Yes	68.2996	Low
P8	706	1	< 60	Male	2	T3N0M0	III + IV	< 400	No	86.4818	High
P9	1254	1	< 60	Female	2	T1N0M0	I + II	≥ 400	Yes	76.6562	Low
P10	354	1	< 60	Male	2	T3N0M0	III + IV	≥ 400	Yes	77.9695	High
P11	763	0	< 60	Male	2	T3N0M0	III + IV	< 400	Yes	80.5424	High
P12	226	1	< 60	Male	2	T3N0M0	III + IV	≥ 400	Yes	86.5067	High
P13	291	1	< 60	Male	2	T2N0M0	I + II	< 400	Yes	98.6736	High
P14	248	1	< 60	Male	2	T4N0M0	-	< 400	Yes	80.3059	High
P15	678	1	< 60	Male	1	T1bN0M0	I + II	< 400	No	89.1674	High
P16	279	1	< 60	Male	2	T3N0M0	III + IV	< 400	Yes	73.5097	Low
P17	510	0	< 60	Male	2	T3N0M0	III + IV	≥ 400	Yes	54.4152	Low
P18	372	1	< 60	Male	2	T3N2M0	III + IV	< 400	Yes	79.8609	High
P19	1314	0	< 60	Male	2	T3N0M0	III + IV	≥ 400	No	77.4180	Low
P20	505	1	< 60	Female	2	T1N0M0	I + II	≥ 400	No	77.7057	High
P21	334	1	< 60	Male	2	T3N1M0	-	≥ 400	No	69.9144	Low
P22	614	1	< 60	Male	2	T3N0M0	III + IV	≥ 400	Yes	64.2983	Low
P23	283	1	< 60	Male	2	T3N1M0	III + IV	≥ 400	Yes	83.7981	High

P24	1181	0	< 60	Female	3	T3N0M0	III + IV	≥ 400	No	77.6121	Low
P25	412	0	< 60	Male	2	T3N0M0	III + IV	< 400	Yes	78.4244	High
P26	842	0	< 60	Female	2	T3N0M0	III + IV	≥ 400	No	70.5079	Low
P27	1145	0	< 60	Male	2	T2N1M0	III + IV	≥ 400	No	27.5804	Low
P28	125	1	< 60	Male	2	T3N0M0	III + IV	< 400	Yes	91.9624	High
P29	198	1	< 60	Male	2	T3N0M0	III + IV	≥ 400	Yes	78.9730	High
P30	869	0	< 60	Female	2	T1bN0M0	I + II	≥ 400	Yes	83.9657	High
P31	498	1	< 60	Female	2	T3N0M0	III + IV	≥ 400	No	69.2045	Low
P32	104	1	< 60	Male	2	T2N0M0	I + II	≥ 400	Yes	73.9779	Low
P33	620	0	< 60	Female	2	T3N0M0	III + IV	< 400	No	75.9721	Low
P34	434	1	< 60	Male	2	T3N0M0	III + IV	< 400	No	61.6751	Low
P35	1211	0	< 60	Male	3	T3N0M0	III + IV	≥ 400	Yes	43.0576	Low
P36	936	1	< 60	Female	3	T3N0M0	III + IV	< 400	Yes	80.3366	High
P37	496	1	< 60	Male	2	T2N1M0	III + IV	< 400	No	74.6776	Low
P38	699	0	< 60	Male	2	T3N0M0	III + IV	< 400	Yes	75.7544	Low
P39	526	0	< 60	Male	2	T3N0M0	III + IV	< 400	No	31.0903	Low
P40	495	1	< 60	Male	2	T1bN0M0	I + II	≥ 400	Yes	89.6480	High
P41	651	1	< 60	Male	2	T3N0M0	III + IV	≥ 400	No	97.8781	High
P42	255	1	< 60	Male	2	T2N0M0	I + II	≥ 400	Yes	133.5352	High
P43	444	1	< 60	Male	2	T1bN0M0	I + II	< 400	Yes	117.1944	High
P44	699	1	< 60	Male	2	T3N0M0	III + IV	< 400	Yes	68.5294	Low
P45	563	1	< 60	Female	2	T2N0M0	I + II	< 400	Yes	88.8244	High
P46	113	1	< 60	Male	2	T3N1M0	III + IV	≥ 400	No	69.3056	Low
P47	771	1	< 60	Female	2	T2N0M0	I + II	≥ 400	No	90.8026	High
P48	944	1	< 60	Male	2	T3N0M0	III + IV	< 400	Yes	88.2134	High

P49	90	1	< 60	Male	2	T3N0M0	III + IV	≥ 400	Yes	79.0614	High
P50	468	1	< 60	Male	2	T2N0M0	I + II	≥ 400	No	96.6409	High
P51	89	1	< 60	Male	3	T3N0M0	III + IV	≥ 400	Yes	61.7195	Low
P52	815	1	< 60	Male	2	T3N0M0	III + IV	≥ 400	Yes	96.1604	High
P53	833	1	< 60	Male	2	T2N0M0	I + II	< 400	Yes	95.7605	High
P54	681	1	< 60	Male	2	T1N0M0	I + II	≥ 400	No	73.3118	Low
P55	366	1	< 60	Male	2	T2N0M0	I + II	≥ 400	Yes	142.5747	High
P56	122	1	< 60	Male	2	T3N0M0	III + IV	≥ 400	Yes	64.8685	Low
P57	342	0	< 60	Male	2	T3N0M0	III + IV	< 400	No	26.6406	Low
P58	373	0	< 60	Male	2	T2N0M0	I + II	< 400	Yes	59.0365	Low
P59	234	1	< 60	Male	2	T2N0M0	I + II	< 400	Yes	65.5478	Low
P60	504	1	< 60	Male	2	T2N0M0	I + II	≥ 400	No	84.4966	High
P61	279	1	< 60	Female	2	T2N0M0	I + II	≥ 400	Yes	76.9519	Low
P62	427	0	< 60	Female	2	T3N0M0	III + IV	< 400	No	66.5019	Low
P63	1272	0	≥ 60	Male	2	T3N0M0	III + IV	≥ 400	No	37.8785	Low
P64	635	0	≥ 60	Male	2	T3N0M0	III + IV	< 400	Yes	25.984	Low
P65	419	1	< 60	Male	3	T3N1M0	III + IV	≥ 400	Yes	73.1484	Low
P66	310	0	< 60	Male	2	T4N0M0	III + IV	< 400	No	72.0211	Low
P67	1532	0	< 60	Female	2	T3N0M0	III + IV	≥ 400	No	76.4428	Low
P68	1174	1	< 60	Male	2	T1bN0M0	I + II	< 400	No	109.2293	High
P69	93	1	≥ 60	Male	2	T2N0M0	I + II	≥ 400	Yes	79.3807	High
P70	674	1	≥ 60	Male	2	T3N0M0	III + IV	< 400	No	98.0508	High
P71	186	1	≥ 60	Male	2	T3N0M0	III + IV	< 400	Yes	96.2477	High
P72	508	1	≥ 60	Male	2	T3N0M0	III + IV	< 400	No	111.0189	High
P73	611	0	≥ 60	Male	2	T2N0M0	I + II	≥ 400	No	35.1047	Low

P74	217	1	≥ 60	Male	1	T3N0M0	III + IV	< 400	Yes	112.119	High
P75	341	0	≥ 60	Female	2	T3N0M0	III + IV	< 400	Yes	33.1375	Low

AFP alpha-fetoprotein, *MELK* maternal embryonic leucine zipper kina

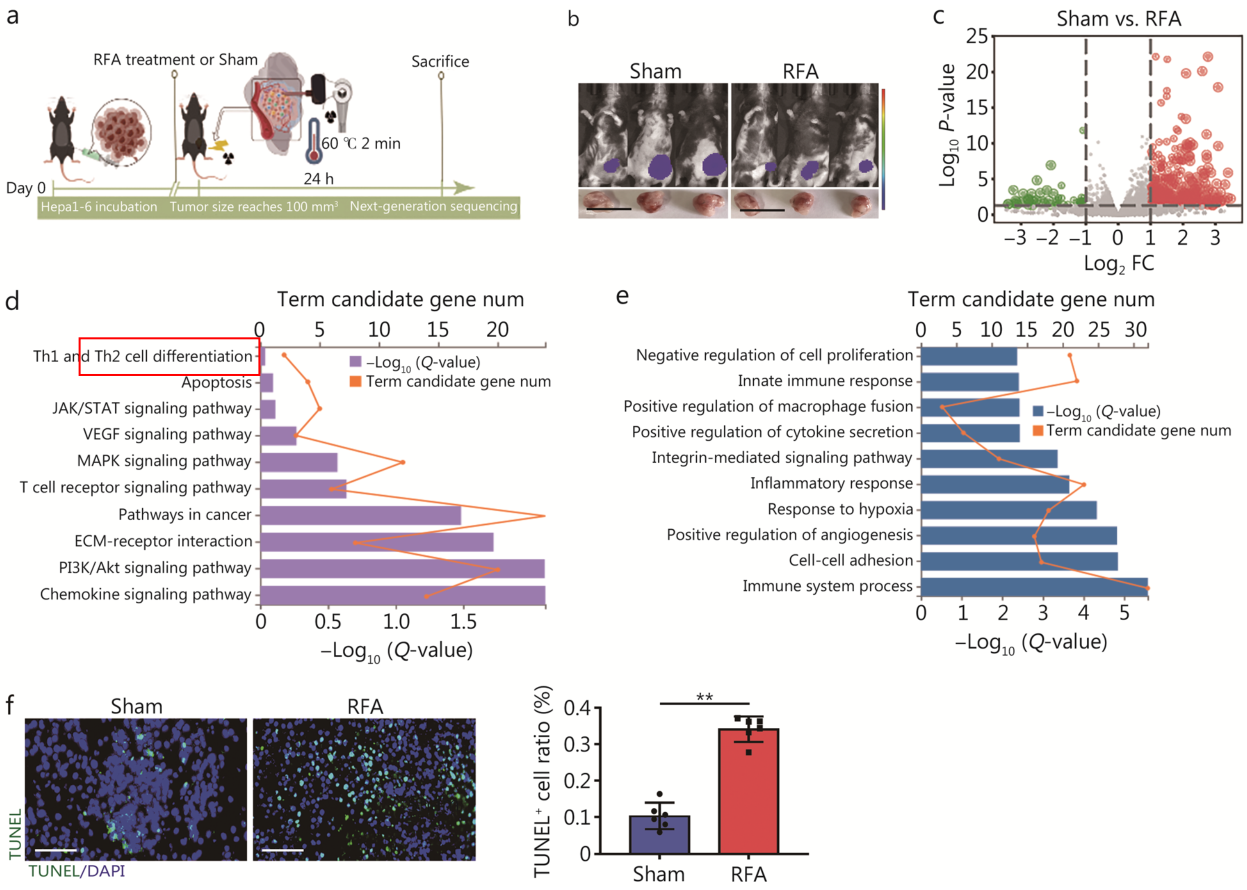

Fig. S1 RFA inhibits tumor progression and induces immune cell infiltration. **a** The flowchart of mouse hepatoma RFA. **b** Sham or RFA of mouse liver tumor model in vivo imaging and tumor isolation diagram (*n* = 3). **c** Volcano plots of DEGs in Hepa1-6 tumor tissues from mice in the sham and RFA treatment groups (*n* = 3). GO (**d**) and KEGG (**e**) enrichment pathway annotations revealed biological processes and signal pathways associated with RFA. **f** IF-based expression levels and corresponding positive ratio of TUNEL in Hepa1-6 tumor tissues. TUNEL is in green, DAPI is in blue. Scale bar = 20 μm. $^{**}P < 0.01$. RFA radiofrequency ablation, FC fold change, DEG differentially expressed gene, GO Gene Ontology, KEGG Kyoto Encyclopedia of Genes and Genomes, DAPI 4′,6-diamidino-2-phenylindole, VEGF vascular endothelial growth factor, MAPK mitogen-activated protein kinases, ECM extracellular matrix, JAK/STAT Janus kinase/signal transducer and activator of transcription, PI3K/Akt phosphatidylinositide 3-kinases/protein kinase B

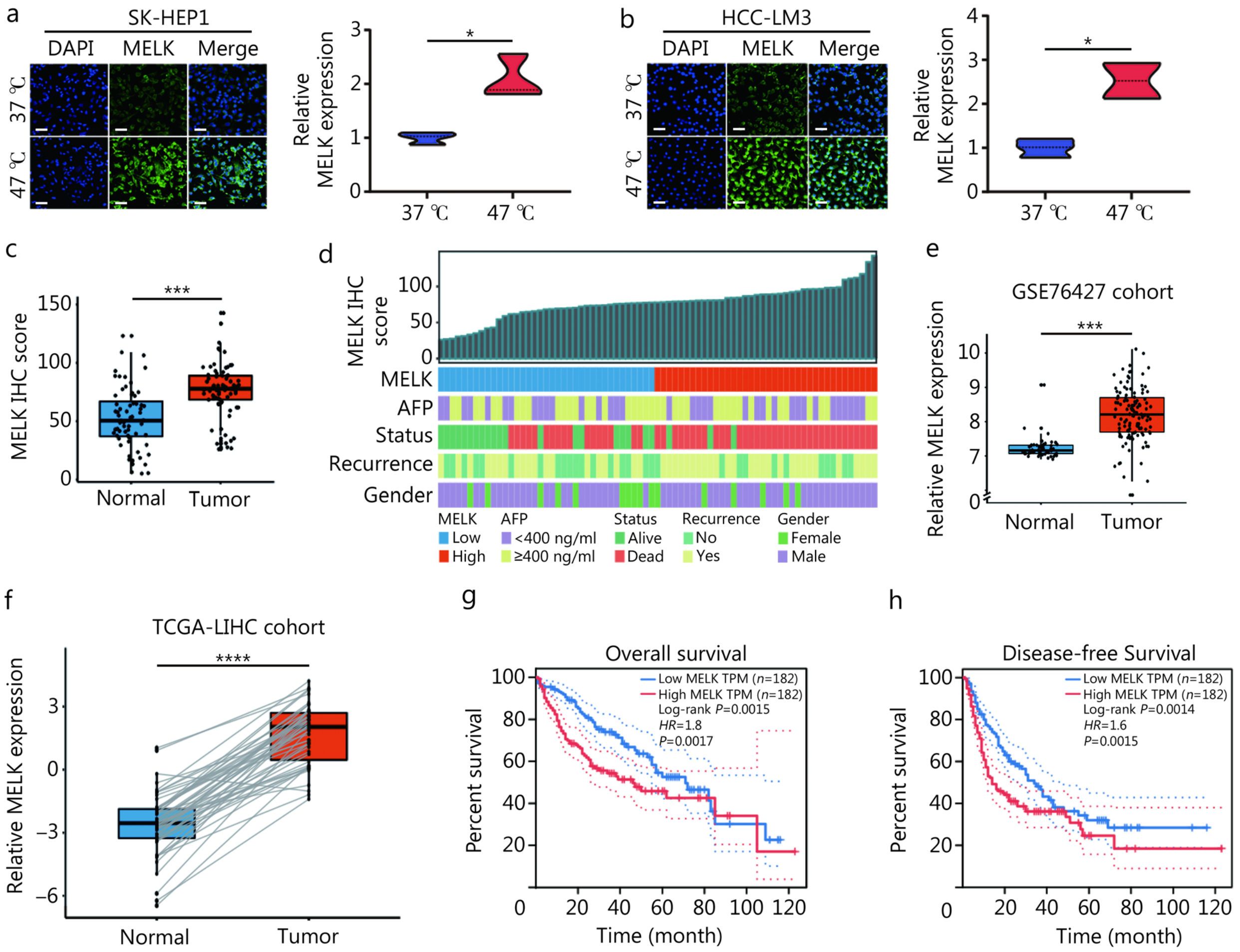

Fig. S2 RFA treatment increased MELK expression, an independent risk factor in patients with HCC. IF images and statistic histograms show MELK expression in SK-HEP1 (**a**) and HCC-LM3 (**b**) cells after hyperthermia treatment. MELK is in green, and DAPI is in blue. **c** IHC statistic shows MELK expression in human liver cancer and adjacent tissues. **d** Graphs of correlation among IHC score of MELK and the expression of AFP, the status of patients, tumor recurrence, and genders of patients. The relative expression of MELK in human liver cancer and adjacent tissues of GSE76427 (GEO database) (**e**) and TCGA-LIHC (**f**) cohorts. **g** Overall survival of patients with liver cancer and different MELK expression in the databases. **h** Disease-free survival of patients with liver cancer and different MELK expression in the databases. Scale bar = 20 μm. $^{*}P < 0.05$, $^{***}P < 0.001$, $^{****}P < 0.0001$. HCC hepatocellular carcinoma, MELK maternal embryonic leucine zipper kinase, TCGA The Cancer Genome Atlas, LIHC liver hepatocellular carcinoma, DAPI 4′,6-diamidino-2-phenylindole, AFP alpha-fetoprotein, GEO Gene Expression Omnibus, TPM transcripts per million

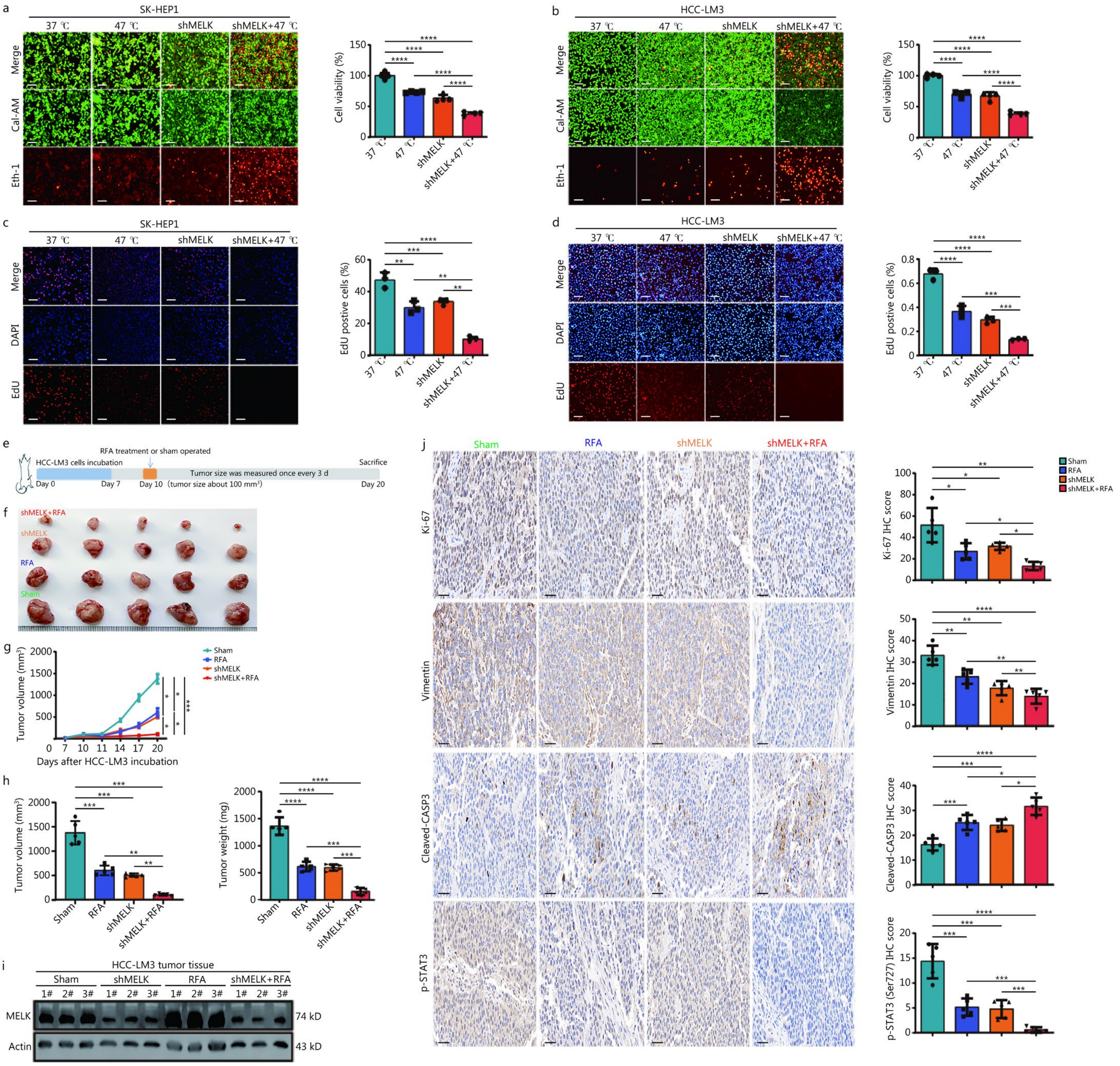

Fig. S3 *MELK* knockdown enhances HCC sensitivity to heat treatment and RFA efficacy. The death of SK-HEP1 (**a**) and HCC-LM3 (**b**) liver cancer cells was detected by calcein AM (Cal-AM) and Eth-1 staining (n = 3). Cal-AM is in green, Eth-1 is in red. The EdU assay detected the survival of SK-HEP1 (**c**) and HCC-LM3 (**d**) cells and corresponding statistic results (n = 3). EdU is in red, DAPI is in blue. **e** Schematic HCC mouse model. HCC-LM3 cells were transplanted subcutaneously into nude mice, and RFA was performed when the tumor volume was about 100 mm^3. **f** An image of HCC-LM3 subcutaneously transplanted tumors in nude mice treated in each group (n = 5). **g** A line graph of HCC-LM3 tumor volumes in different groups. **h** The tumor volume and weight of HCC-LM3 subcutaneous transplanted tumors in nude mice. **i** The expression of MELK in tumor tissues of the four groups. **j** Representative images of IHC for Ki-67, Vimentin, cleaved-CASP3, and p-STAT3 (Ser727) in HCC-LM3 tumor tissues. Scale bar = 20 μm. $^*P$ < 0.05, $^{**}P$ < 0.01, $^{***}P$ < 0.001, $^{****}P$ < 0.0001. MELK maternal embryonic leucine zipper kinase, DAPI 4′,6-diamidino-2-phenylindole, RFA radiofrequency ablation, HCC hepatocellular carcinoma, IHC immunohistochemistry, p-STAT3 phosphorylation of the transcription factor signal transducer and activator

of transcription 3, CASP3 caspase-3

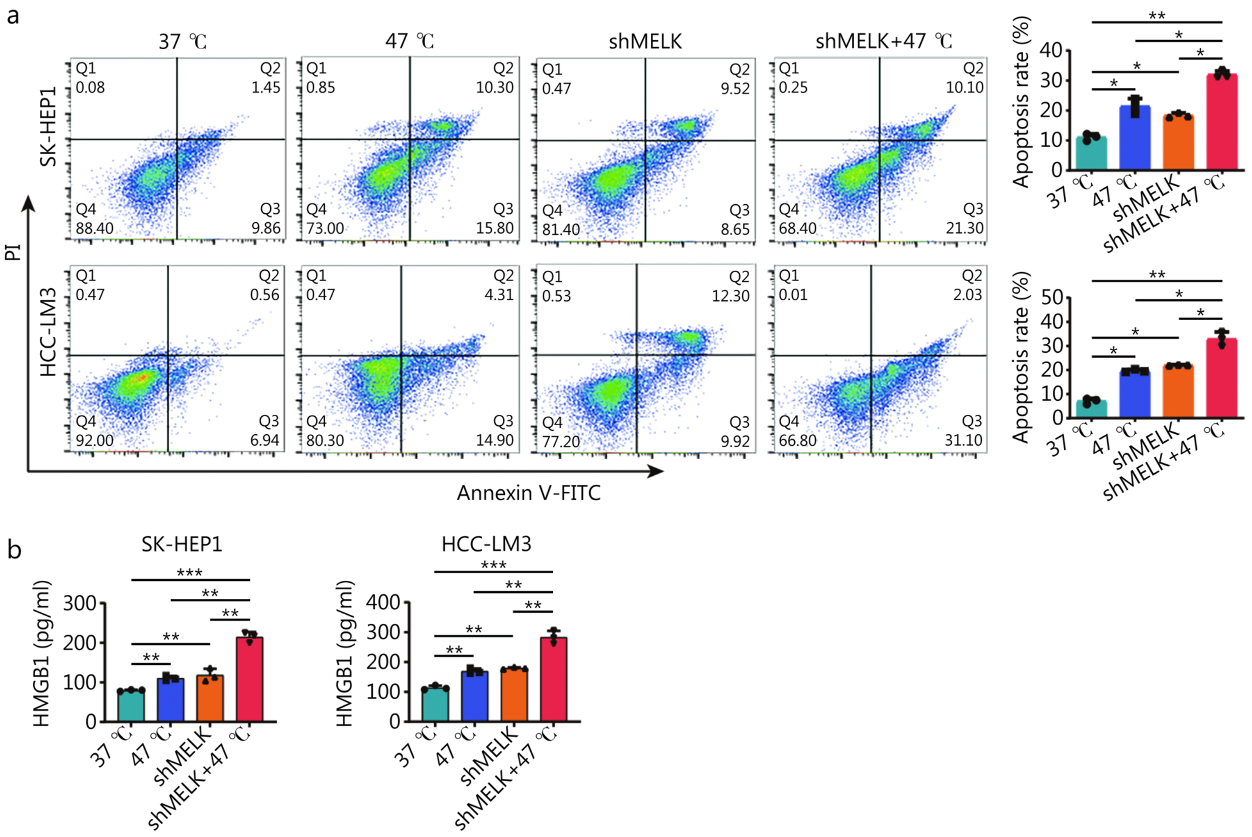

Fig. S4 *MELK* knockdown promotes RFA-induced apoptosis and immunogenic death in hepatoma cells. **a** Flow cytometry and statistic histograms show the apoptosis of SK-HEP1 and HCC-LM3 liver cancer cells (n = 3). **b** Enzyme-linked immunosorbent assays (ELISA) results show the secretion level of HMGB1 in SK-HEP1 and HCC-LM3 cells (n = 3). $^{*}P < 0.05$, $^{**}P < 0.01$, $^{***}P < 0.001$. MELK maternal embryonic leucine zipper kinase, HMGB1 high mobility group box 1

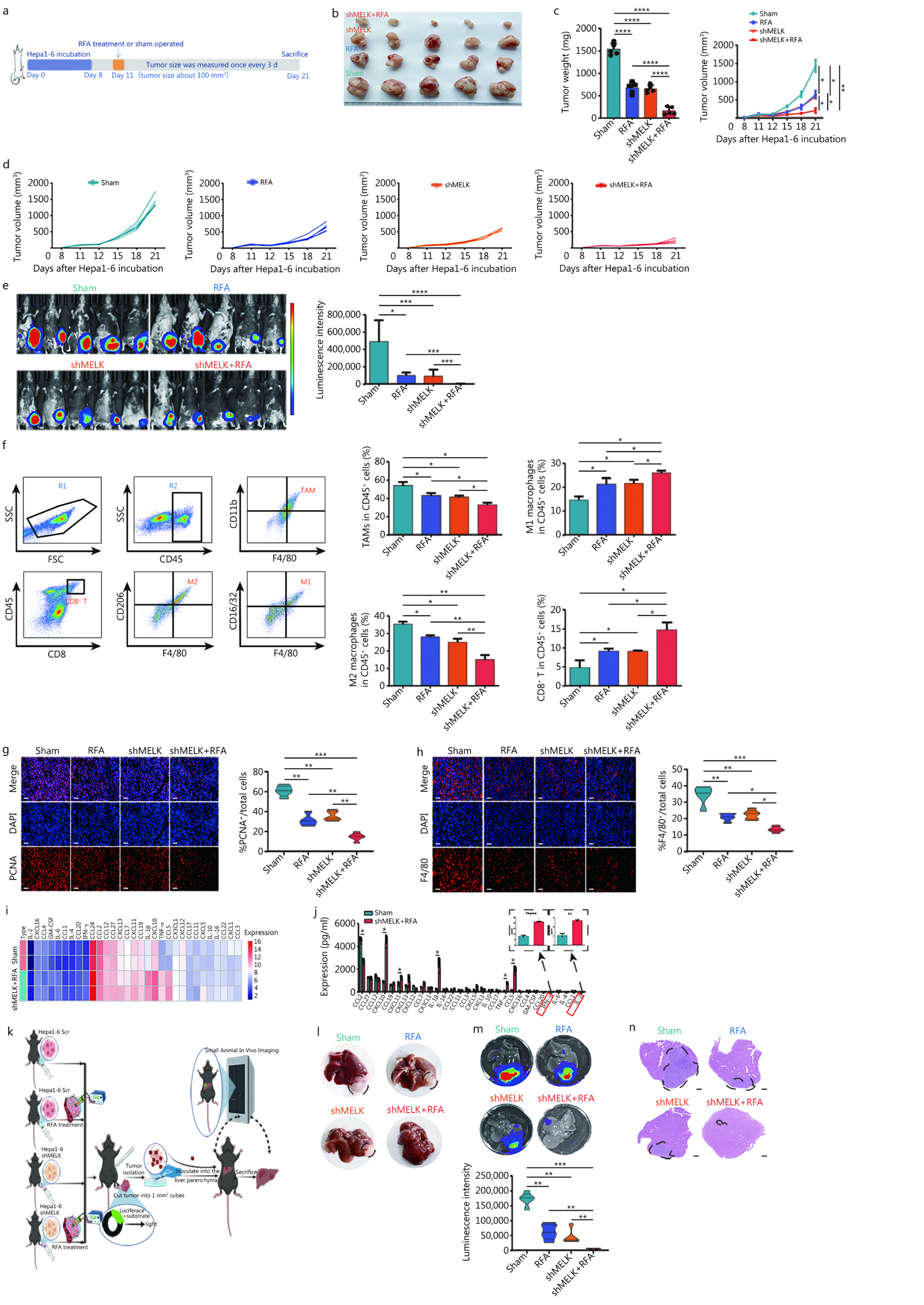

Fig. S5 *MELK* knockdown enhances RFA-induced antitumor immune effects in HCC. **a** Schematic diagram

of model construction. Hepa1-6 cells were subcutaneously transplanted into C57BL/6 mice, and RFA was performed when the tumor volume reached about 100 mm^3. **b** Image of a Hepa1-6 subcutaneous transplanted tumor in each group (*n* = 5). **c** The weights and volumes of Hepa1-6 tumors. **d** The volumes of Hepa1-6 tumors in single mice of the 4 groups. **e** Fluorescence intensity of Hepa1-6 subcutaneous grafted tumors monitored with an IVIS imaging system. Fluorescence images of view were captured at 21 d subsequent to implantation and D-luciferin injection. The color bar is on the right of the Fluorescence images. Quantitative fluorescence analysis of small animal live imaging (*n* = 5). **f** It shows the corresponding sorting policy of TAMs, M1 macrophages, M2 macrophages, and $CD8^+$ T cells**.** Quantitative CytoFlow analysis of TAMs, M1 macrophages, M2 macrophages, and $CD8^+$ T cells (*n* = 5). Representative IF images and fluorescence statistics of proliferating cell nuclear antigen (PCNA; **g**) and F4/80 (**h**) expression in tumor tissues of the Hepa1-6 subcutaneous graft models. Scale bar = 20 μm. PCNA is in red, F4/80 is in red, DAPI is in blue (*n* = 5). **i** Multiplex cytokine array shows the cytokines secreted by liver cancer. **j** The secretion statistics of cytokines detected by the multiplex cytokine assay. **k** The flowchart of the Hepa1-6 orthotopic tumor model. **l** A representative image of the Hepa1-6 orthotopic tumor model (*n* = 5). **m** A representative image of the Hepa1-6 orthotopic tumor model with the IVIS imaging system. Luminescence intensity statistics of the Hepa1-6 orthotopic tumor model. **n** A representative hematoxylin and eosin (HE) image of the Hepa1-6 orthotopic tumor model. Scale bar = 5 mm. $^{*}P < 0.05$, $^{**}P < 0.01$, $^{***}P < 0.001$, $^{****}P < 0.0001$. RFA radiofrequency ablation, MELK maternal embryonic leucine zipper kinase, DAPI 4′,6-diamidino-2-phenylindole, HCC hepatocellular carcinoma, IVIS In Vivo Imaging System, Scr scrambled shRNA, GM-CSF granulocyte-macrophage colony-stimulating factor, TNF-α tumor necrosis factor-α, IL-1β interleukin-1β, IL-2 interleukin-2, IL-4 interleukin-4, IL-6 interleukin-6, IL-10 interleukin-10, IL-16 interleukin-16, INF-γ interferon-gamma, CCL1 chemokine (C-C motif) ligand 1, CCL3 chemokine (C-C motif) ligand 3, CCL4 chemokine (C-C motif) ligand 4, CCL-5 chemokine (C-C motif) ligand 5, CCL-7 chemokine (C-C motif) ligand 7, CCL-12 chemokine (C-C motif) ligand 12, CCL17 chemokine (C-C motif) ligand 17, CCL19 chemokine (C-C motif) ligand 19, CCL20 chemokine (C-C motif) ligand 20, CCL22 chemokine (C-C motif) ligand 22, CCL-24 chemokine (C-C motif) ligand 24, CX3CL1 C-X3-C motif chemokine ligand 1, CXCL1 C-X-C motif chemokine ligand 1, CXCL10 C-X-C motif chemokine ligand 10, CXCL11 C-X-C motif chemokine ligand 11, CXCL12 C-X-C motif chemokine ligand 12, CXCL13 C-X-C motif chemokine ligand 13, CXCL15 C-X-C motif chemokine ligand 15, CXCL16 C-X-C motif chemokine ligand 16

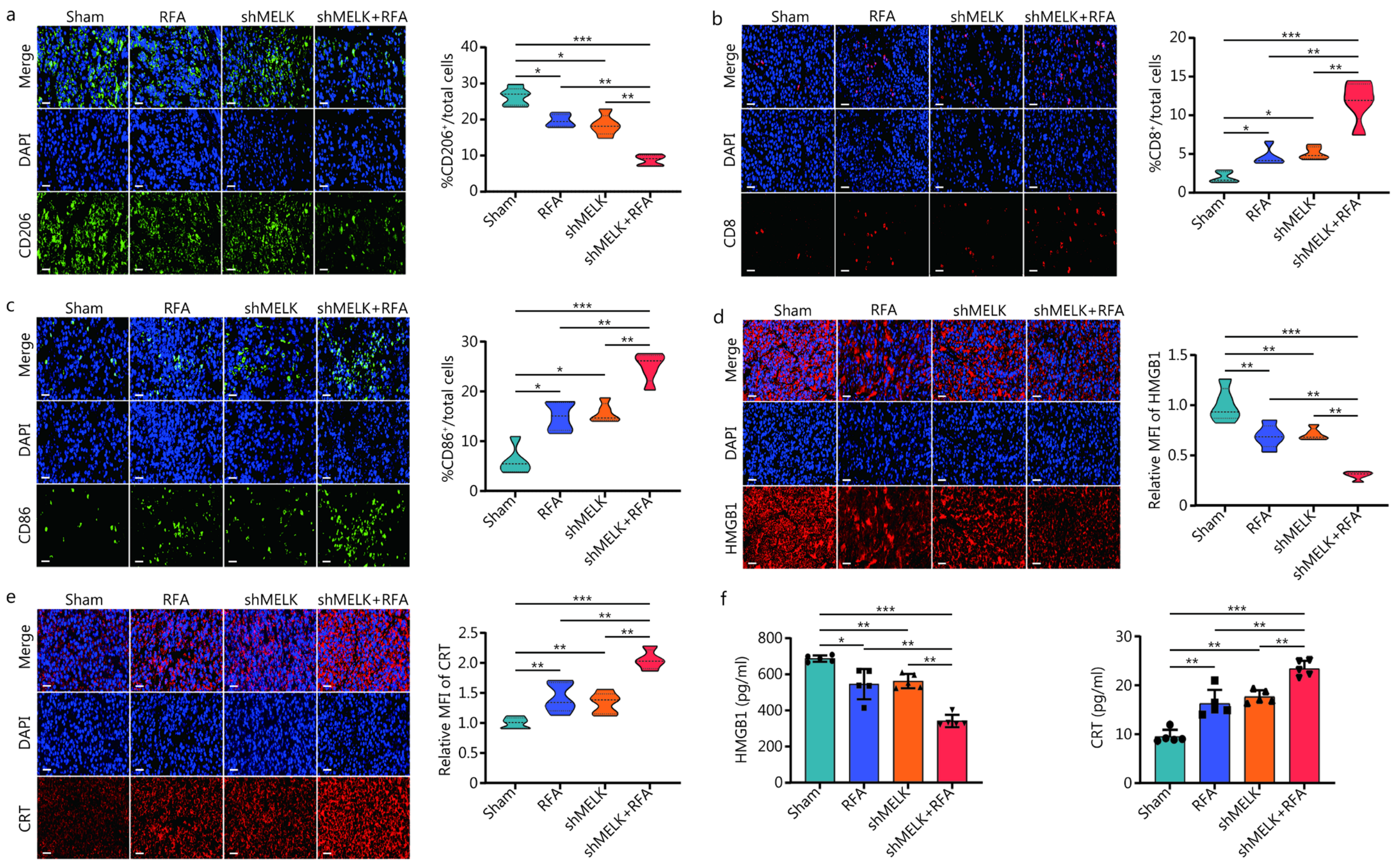

Fig. S6 The changes in immune cell infiltration in liver tumors after RFA treatment or *MELK* knockdown. Flow cytometry analysis of TAMs, M1 macrophage, M2 macrophage, and $CD8^+$ T cell infiltration into tumor tissues of Hepa1-6 subcutaneous tumor bearing model. Representative IF images and percentages of CD206 (**a**), CD8 (**b**), and CD86 (**c**) in Hepa1-6 tumor tissue with different treatments (n = 5), including sham, RFA, shMELK, and shMELK + RFA. CD206 is in green, CD8 is in red, CD86 is in green, DAPI is in blue. Representative IF images and relative MFI of HMGB1 (**d**) and CRT (**e**) in Hepa1-6 tumor tissue with different treatments (n = 5). HMGB1 is in red, CRT is in red, DAPI is in blue. **f** The concentration of HMGB1 and CRT in Hepa1-6 tumor tissue (n = 5) by enzyme-linked immunosorbent assays (ELISA). Scale bar = 20 μm. $^*P < 0.05$, $^{**}P < 0.01$, $^{***}P < 0.001$. R1 Regin1, R2 Regin2, SSC side scatter, FSC forward scatter, TAM tumour-associated macrophages, RFA radiofrequency ablation, MELK maternal embryonic leucine zipper kinase, IF immunofluorescence, DAPI 4′,6-diamidino-2-phenylindole, HMGB1 high mobility group box 1, CRT Calreticulin, MFI mean fluorescence intensity

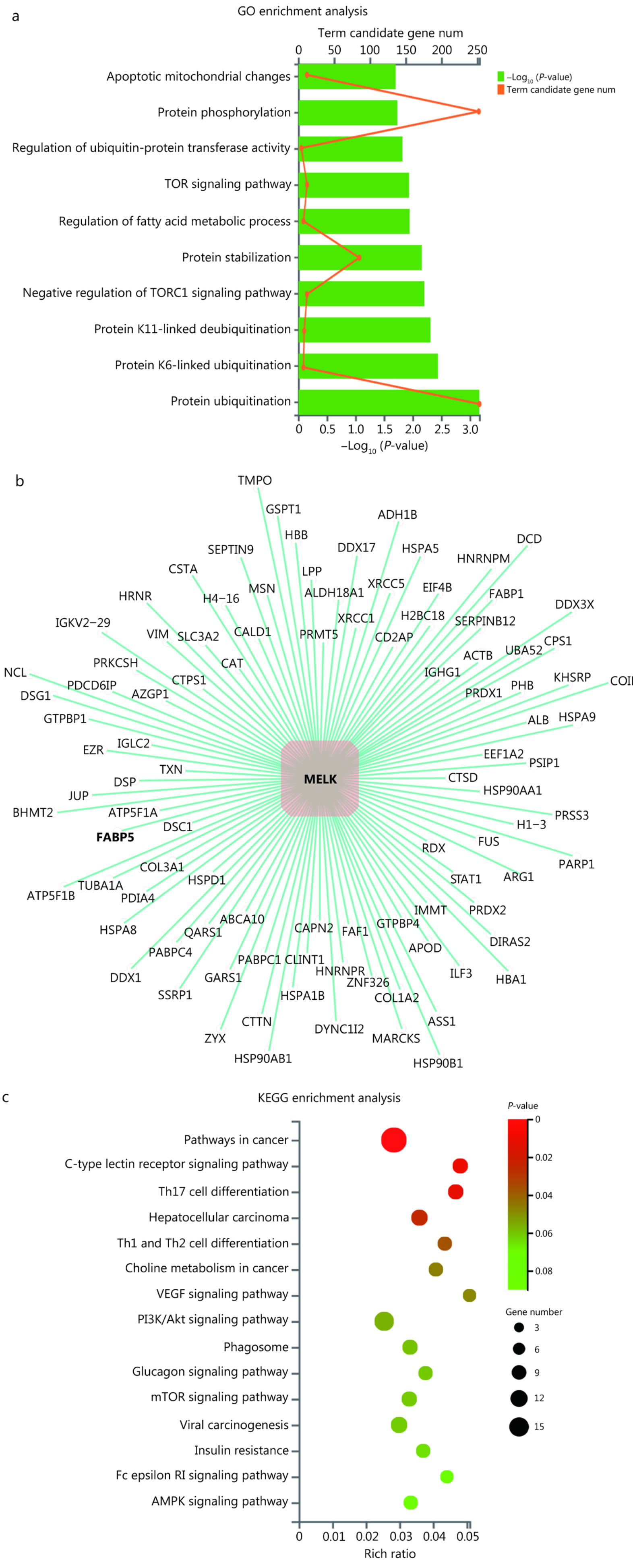

Fig. S7 Tumor cell-intrinsic MELK enhanced the PI3K/Akt/mTOR signal axis and FABP5 interaction. **a** GO enrichment analysis shows *MELK* knockdown and hyperthermia co-treatment-related signaling pathways in

SK-HEP1 cells. **b** A schematic diagram of proteins interacting with MELK protein by liquid chromatography-mass spectrometry (LC-MS). **c** KEGG enrichment analysis of the signaling pathway of MELK-interacting proteins. MELK maternal embryonic leucine zipper kinase, GO Gene Ontology, KEGG Kyoto Encyclopedia of Genes and Genomes, PI3K phosphoinositide 3-kinase, Akt protein kinase B, mTOR mammalian target of rapamycin, TOR target of rapamycin, TORC1 target of rapamycin complex 1, VEGF vascular endothelial growth factor, AMPK adenosine 5'-monophosphate (AMP)-activated protein kinase

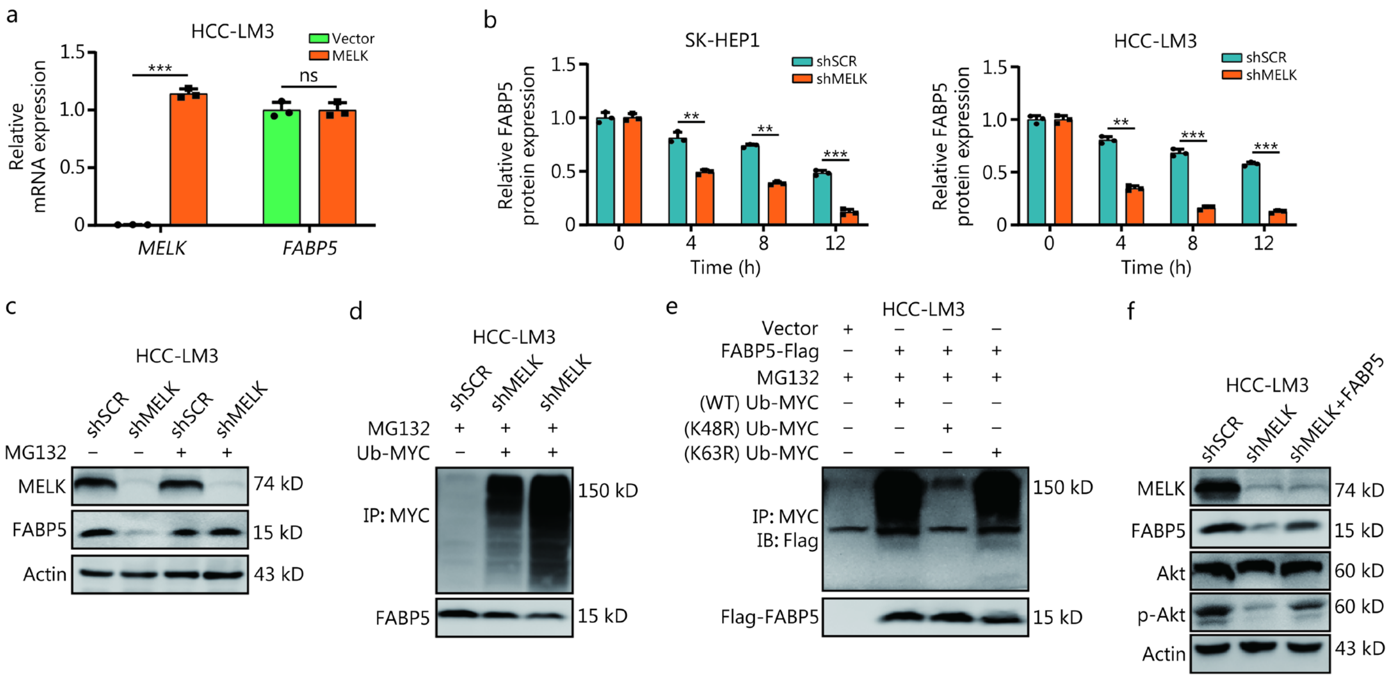

Fig. S8 MELK decreased the Ub level of FABP5 to maintain its stability. **a** qPCR shows the relative mRNA expression of *MELK* and *FABP5* in HCC-LM3 cells. **b** Enzyme-linked immunosorbent assays (ELISA) show FABP5 expression in SK-HEP1 and HCC-LM3 cells after CHX treatment at 0, 4, 8, and 12 h. **c** Western blotting shows FABP5 expression in HCC-LM3 cells at MG132 treatment or not. **d** Western blotting shows Ub expression in HCC-LM3 cells with or without *MELK* knockdown after MG132 treatment. **e** Western blotting shows Ub-K48R and Ub-K63R levels in HCC-LM3 cells. **f** Western blotting shows MELK, FABP5, and Akt/p-Akt levels in HCC-LM3 hepatoma cells. $^{**}P < 0.01$, $^{***}P < 0.001$, ns not significant. MELK maternal embryonic leucine zipper kinase, Ub ubiquitination, FABP5 fatty acid-binding protein 5, PKD protein kinase domain, MYC V-myc avian myelocytomatosis viral oncogene homolog, CHX chlorhexidine, Akt protein kinase B, SCR scramble, MG132 Z-Leu-Leu-Leu-al

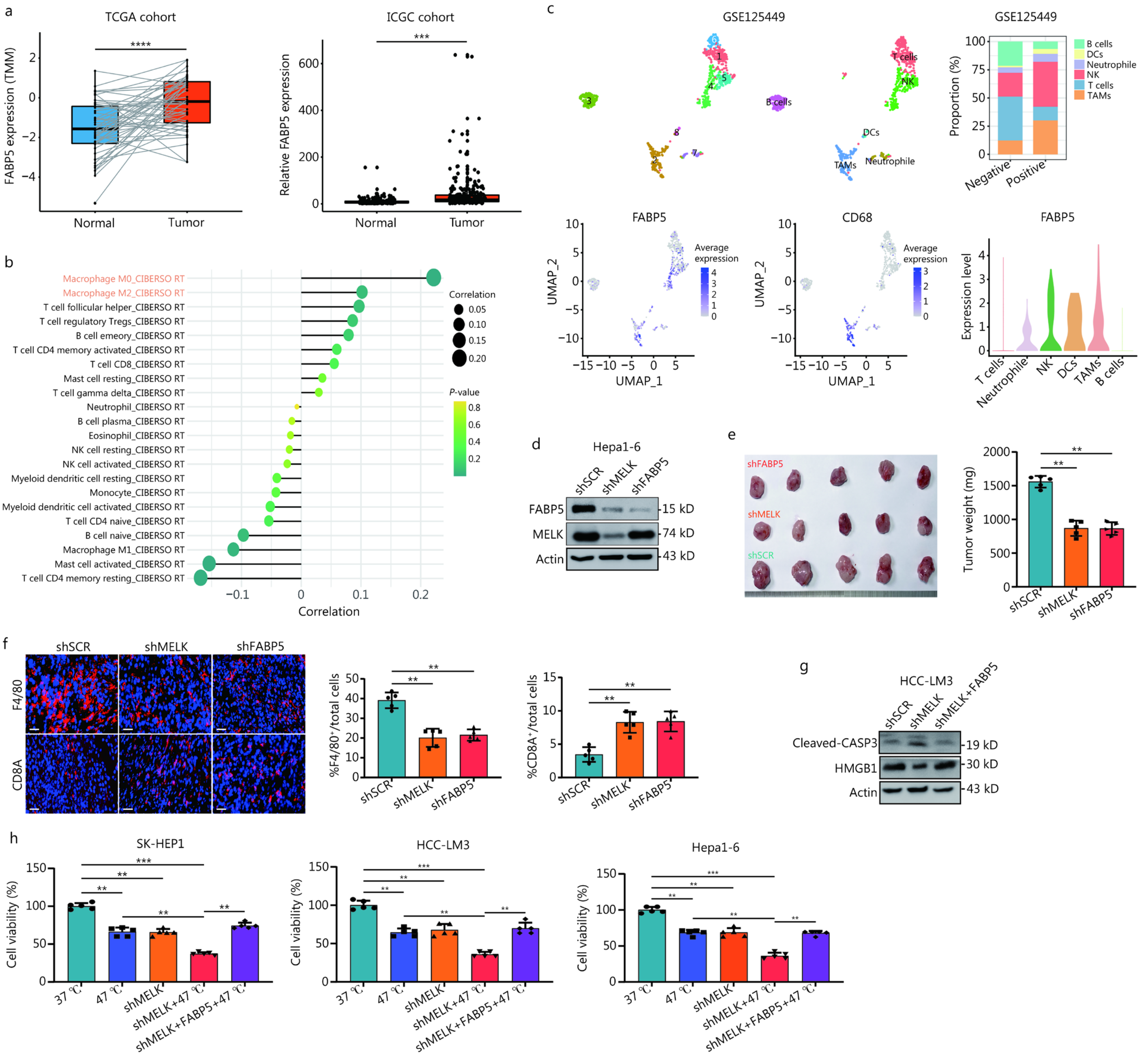

Fig. S9 FABP5 is required for the antitumor effect of RFA treatment and *MELK* knockdown in HCC. **a** The TCGA and ICGC databases were used to verify FABP5 expression in HCC and adjacent tissues. **b** CIBERSORT analyses quantifying 22 immune cell subtypes and overall inferred immune infiltrate in HCC tumors. **c** Single-cell RNA-sequencing analysis of the GSE125449 dataset to identify immune cell types infiltrating HCC. UMAP clustering of different immune cell clusters and percentages of FABP5-positive or -negative positions or cell types. UMAP analysis shows the high expression clusters for FABP5 or CD68$^+$. Violin plots show FABP5 expression in different immune cell clusters. **d** Western blotting shows the expression of MELK, FABP5, and actin in Hepa1-6 cell lines after *MELK* or *FABP5* knockdown. **e** An image shows a Hepa1-6 subcutaneous transplantation tumor at day 21, Hepa1-6 cell lines with *FABP5*- or *MELK*-knockdown (n = 5). **f** Representative IF images show the F4/80 or CD8A in Hepa1-6 tumor tissues. And the percentage of F4/80$^+$ or CD8A$^+$ cells in total cells (n = 5). **g** Western blotting shows cleaved-CASP3, HMGB1, and actin levels in HCC-LM3 cells. **h** The CCK-8 analysis shows the viability of SK-HEP1, HCC-LM3, and Hepa1-6 cells with different treatments. Scale bar = 20 µm. $^{**}P < 0.01$, $^{***}P < 0.001$. TCGA The Cancer Genome Atlas, ICGC International Cancer Genome Consortium, FABP5 fatty acid-binding

protein 5, GSEA gene set enrichment analysis, UMAP uniform manifold approximation and projection, MELK maternal embryonic leucine zipper kinase, IF immunofluorescence.

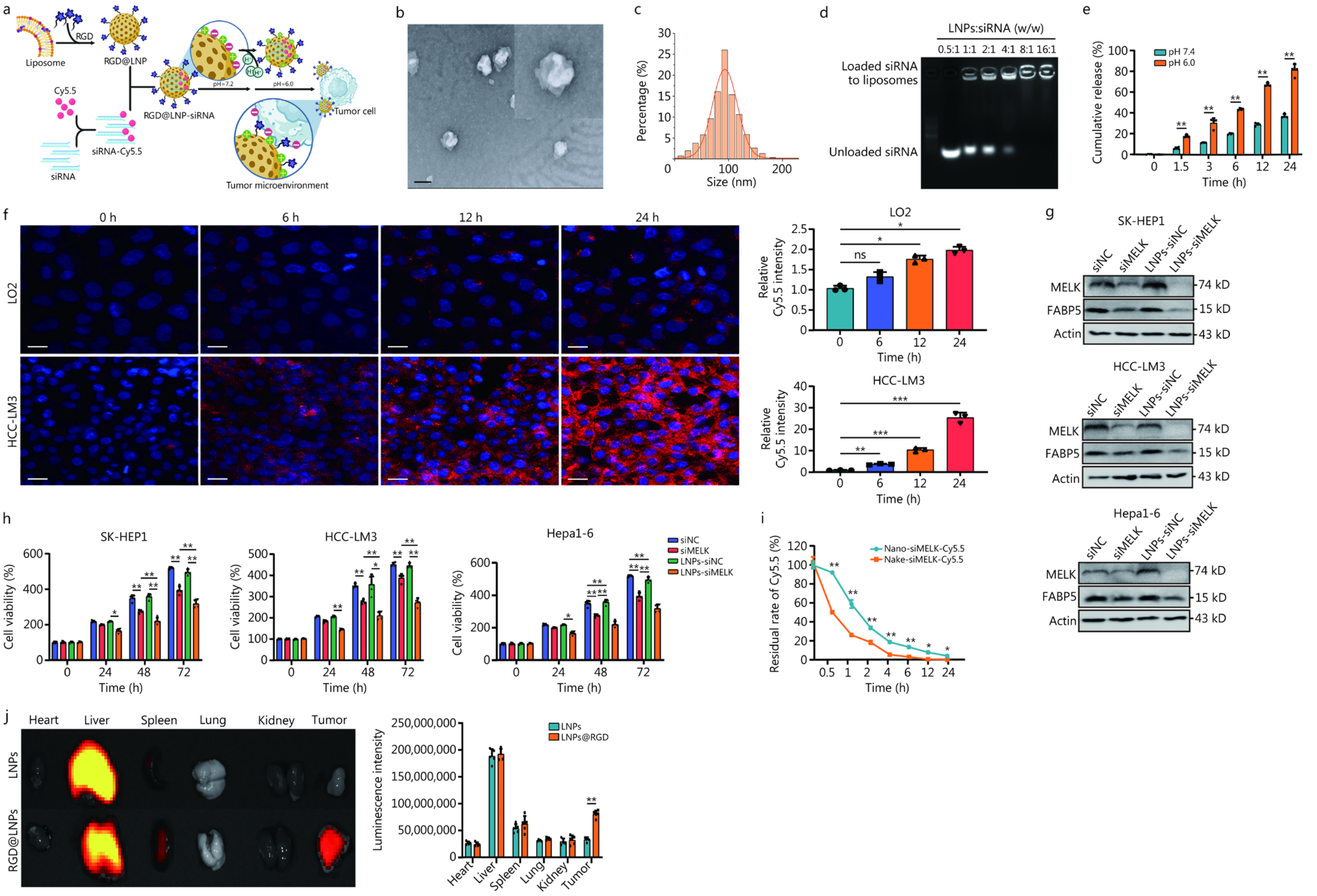

Fig. S10 LNPs with RGD-MELK-siRNAs were synthesized. **a** Schematic representation of the construction of the RGD-LNP-siMELK-Cy5.5. **b** A TEM image of LNPs showing particles with a 100 nm diameter. **c** The size distribution of LNPs. **d** Electrophoretic analysis of LNPs-siRNAs at various mass ratios. **e** The cumulative release percentage of siMELK in different pH environments. **f** Representative IF images of LNPs uptake in LO2 normal liver cells and HCC-LM3 liver cancer cells. A histogram of relative Cy5.5 intensities in LO2 liver cells and HCC-LM3 liver cancer cells at different times (n = 3). **g** MELK, FABP5, and actin expression in SK-HEP1, HCC-LM3, and Hepa1-6 cells after different treatments, including siNC and siMELK, LNPs-siNC, and LNPs-siMELK. **h** The viability of SK-HEP1, HCC-LM3, and Hepa1-6 cells with different treatments. **i** Fluorescence detection of the percentage of residual Cy5.5 in the serum with time when Nano-siMELK-Cy5.5 or Naked-siMELK-Cy5.5 are applied. **j** Fluorescence optical images show Cy5.5 accumulations in different tissues when RGD-LNPs or LNPs are applied. A histogram of the luminescence intensity in different organs when RGD-LNPs or LNPs are applied (n = 5). Scale bar = 20 μm. $^{*}P < 0.05$, $^{**}P < 0.01$, $^{***}P < 0.001$, ns not significant. RGD arginylglycylaspartic acid, LNP lipid nanoparticle, MELK maternal embryonic leucine zipper kinase, FABP5 fatty acid-binding protein 5, TEM transmission electron microscope

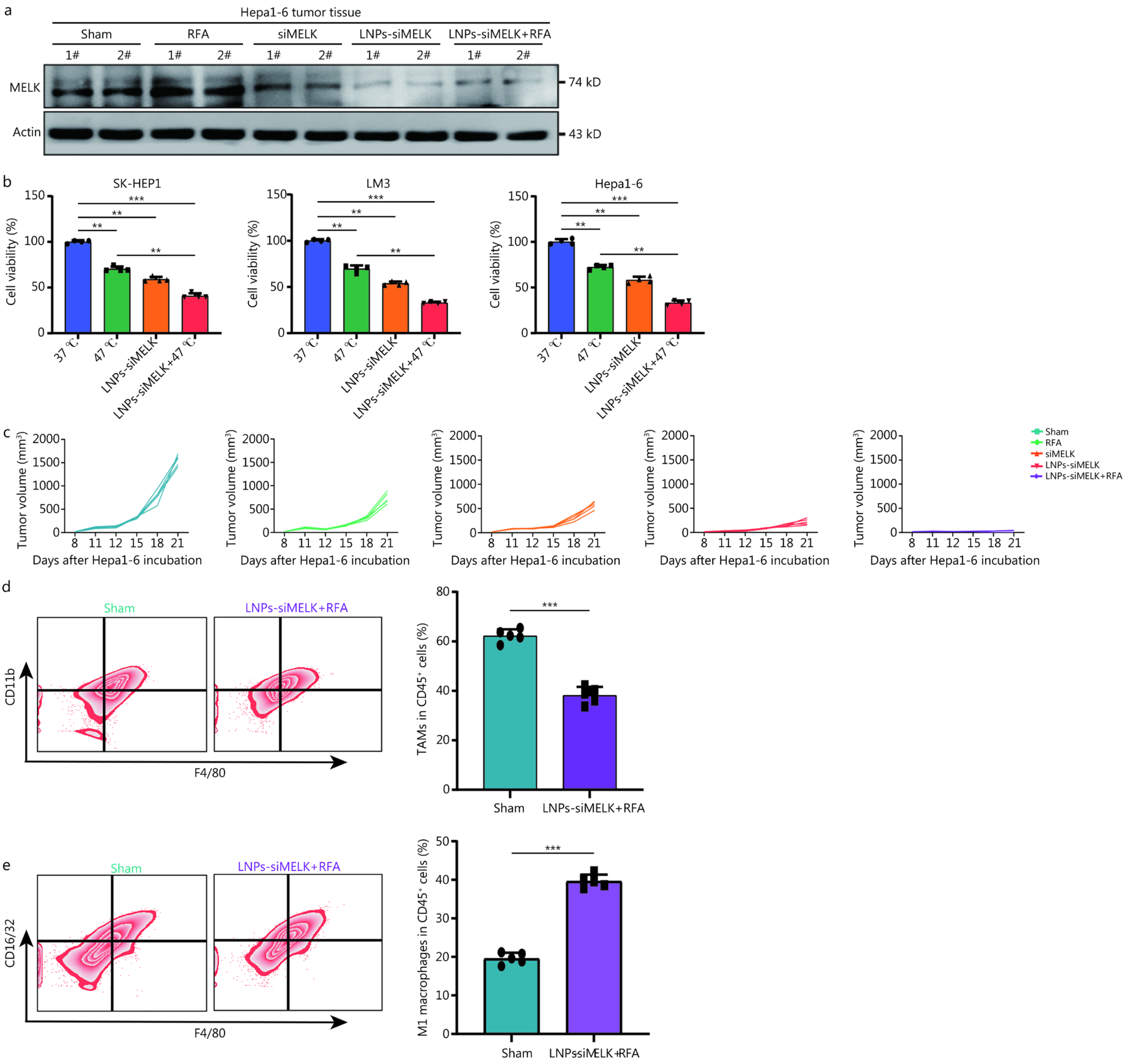

Fig. S11 LNPs-siRNAs targeting tumor cell-intrinsic MELK enhance RFA-induced antitumor immune effects in HCC. **a** The expression of MELK after the combination treatment. Western blotting shows MELK and actin expression in Hepa1-6 cells with different treatments, including sham, RFA, siMELK, LNPs-siMELK, and LNPs-siMELK + RFA. **b** The CCK-8 assay shows the viability of SK-HEP1, HCC-LM3, and Hepa1-6 cells. **c** The monitoring data shows Hepa1-6 subcutaneous tumor growth in each mouse. Flow cytometry analysis and percentages of TAM cells (**d**) and M1 macrophages (**e**) in Hepa1-6 tumor tissue (n = 5). $^{**}P < 0.01$, $^{***}P < 0.001$. RFA radiofrequency ablation, LNP lipid nanoparticle, MELK maternal embryonic leucine zipper kinase, TAM tumour-associated macrophages

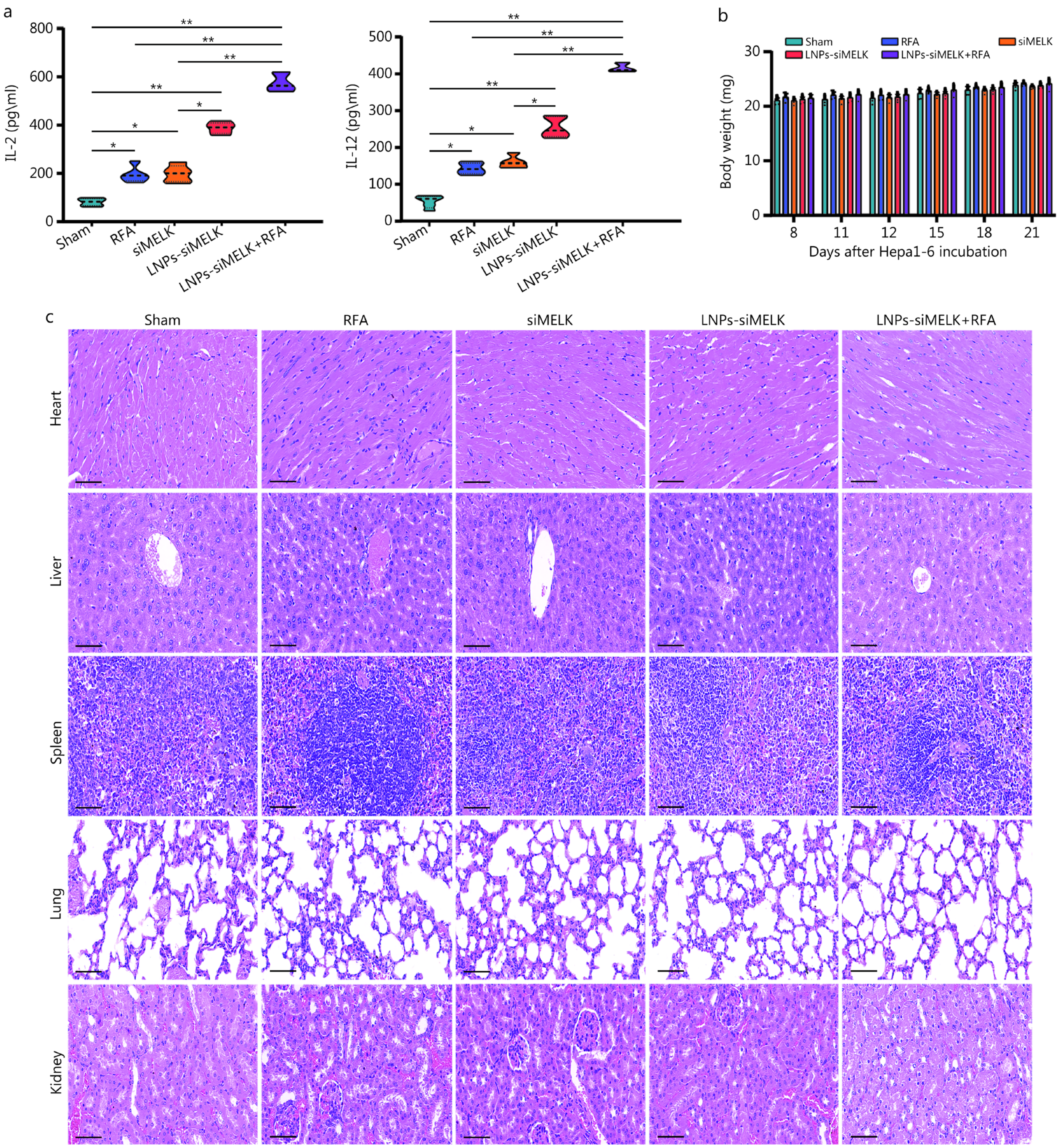

Fig. S12 The immune effect and therapeutic toxicity detection. **a** Enzyme-linked immunosorbent assays (ELISA)-based expression levels of IL-2 and IL-12 in liver tumors with different treatments. **b** A histogram of mouse body weights every three days from day 8 until the mice were euthanized. **c** Representative HE images of the heart, liver, spleen, lung, and kidney in the different treatment groups. Scale bar = 20 μm. $^{*}P < 0.05$, $^{**}P < 0.01$. RFA radiofrequency ablation, LNP lipid nanoparticle, MELK maternal embryonic leucine zipper kinase
